# A Genome-Wide Association Study Identifies Protein Quantitative Trait Loci (pQTLs)

**DOI:** 10.1371/journal.pgen.1000072

**Published:** 2008-05-09

**Authors:** David Melzer, John R. B. Perry, Dena Hernandez, Anna-Maria Corsi, Kara Stevens, Ian Rafferty, Fulvio Lauretani, Anna Murray, J. Raphael Gibbs, Giuseppe Paolisso, Sajjad Rafiq, Javier Simon-Sanchez, Hana Lango, Sonja Scholz, Michael N. Weedon, Sampath Arepalli, Neil Rice, Nicole Washecka, Alison Hurst, Angela Britton, William Henley, Joyce van de Leemput, Rongling Li, Anne B. Newman, Greg Tranah, Tamara Harris, Vijay Panicker, Colin Dayan, Amanda Bennett, Mark I. McCarthy, Aimo Ruokonen, Marjo-Riitta Jarvelin, Jack Guralnik, Stefania Bandinelli, Timothy M. Frayling, Andrew Singleton, Luigi Ferrucci

**Affiliations:** 1 Department of Epidemiology and Public Health, Institute of Biomedical and Clinical Sciences, Peninsula College of Medicine and Dentistry, University of Exeter, Devon, United Kingdom; 2 Genetics of Complex Traits, Institute of Biomedical and Clinical Sciences, Peninsula College of Medicine and Dentistry, University of Exeter, Devon, United Kingdom; 3 Laboratory of Neurogenetics, National Institute of Aging, Porter Neuroscience Research Center, Bethesda, Maryland, United States of America; 4 Tuscany Regional Health Agency, I.O.T. and Department of Medical and Surgical Critical Care, University of Florence, Florence, Italy; 5 Department of Geriatric Medicine and Metabolic Diseases, Second University of Naples, Naples, Italy; 6 School of Mathematics and Statistics, University of Plymouth, Plymouth, United Kingdom; 7 Department of Preventive Medicine and Center for Genomics and Bioinformatics, College of Medicine, University of Tennessee Health Science Center, Memphis, Tennessee, United States of America; 8 University of Pittsburgh, Graduate School of Public Health, Departments of Epidemiology and Medicine, Pittsburgh, Pennsylvania, United States of America; 9 San Francisco Coordinating Center, California Pacific Medical Center Research Institute, San Francisco, California, United States of America; 10 Laboratory of Epidemiology, Demography and Biometry, National Institute on Aging, Bethesda, Maryland, United States of America; 11 Henry Wellcome Laboratories for Integrative Neurosciences and Endocrinology, University of Bristol, Bristol, United Kingdom; 12 Oxford Centre for Diabetes, Endocrinology and Metabolism, Headington, Oxford, United Kingdom; 13 The Wellcome Trust Centre for Human Genetics, Roosevelt Drive, Oxford, United Kingdom; 14 Department of Clinical Chemistry, University of Oulu, Oulu, Finland; 15 Department of Public Health, Science, and General Practice, University of Oulu, Oulu, Finland; 16 Department of Epidemiology and Public Health, Imperial College London, London, United Kingdom; 17 Geriatric Unit, Azienda Sanitaria di Firenze, Florence, Italy; 18 Longitudinal Studies Section, Clinical Research Branch, Gerontology Research Center, National Institute on Aging, Baltimore, Maryland, United States of America; University of Pennsylvania, United States of America

## Abstract

There is considerable evidence that human genetic variation influences gene expression. Genome-wide studies have revealed that mRNA levels are associated with genetic variation in or close to the gene coding for those mRNA transcripts – *cis* effects, and elsewhere in the genome – *trans* effects. The role of genetic variation in determining protein levels has not been systematically assessed. Using a genome-wide association approach we show that common genetic variation influences levels of clinically relevant proteins in human serum and plasma. We evaluated the role of 496,032 polymorphisms on levels of 42 proteins measured in 1200 fasting individuals from the population based InCHIANTI study. Proteins included insulin, several interleukins, adipokines, chemokines, and liver function markers that are implicated in many common diseases including metabolic, inflammatory, and infectious conditions. We identified eight *Cis* effects, including variants in or near the *IL6R* (p = 1.8×10^−57^), *CCL4L1* (p = 3.9×10^−21^), *IL18* (p = 6.8×10^−13^), *LPA* (p = 4.4×10^−10^), *GGT1* (p = 1.5×10^−7^), *SHBG* (p = 3.1×10^−7^), *CRP* (p = 6.4×10^−6^) and *IL1RN* (p = 7.3×10^−6^) genes, all associated with their respective protein products with effect sizes ranging from 0.19 to 0.69 standard deviations per allele. Mechanisms implicated include altered rates of cleavage of bound to unbound soluble receptor (*IL6R*), altered secretion rates of different sized proteins *(LPA),* variation in gene copy number *(CCL4L1)* and altered transcription *(GGT1)*. We identified one novel *trans* effect that was an association between ABO blood group and tumour necrosis factor alpha (TNF-alpha) levels (p = 6.8×10^−40^), but this finding was not present when TNF-alpha was measured using a different assay , or in a second study, suggesting an assay-specific association. Our results show that protein levels share some of the features of the genetics of gene expression. These include the presence of strong genetic effects in *cis* locations. The identification of protein quantitative trait loci (pQTLs) may be a powerful complementary method of improving our understanding of disease pathways.

## Author Summary

One of the central dogmas of molecular genetics is that DNA is transcribed to RNA which is translated to protein and alterations to proteins can influence human diseases. Genome-wide association studies have recently revealed many new DNA variants that influence human diseases. To complement these efforts, several genome-wide studies have established that DNA variation influences mRNA expression levels. Loci influencing mRNA levels have been termed “eQTLs”. In this study we have performed the first genome-wide association study of the third piece in this jigsaw – the role of DNA variation in relation to protein levels, or “pQTLs”. We analysed 42 proteins measured in blood fractions from the InCHIANTI study. We identified eight *cis* effects including common variants in or near the *IL6R*, *CCL4*, *IL18*, *LPA*, *GGT1*, *SHBG*, *CRP* and *IL1RN* genes, all associated with blood levels of their respective protein products. Mechanisms implicated included altered transcription *(GGT1)* but also rates of cleavage of bound to unbound soluble receptor (*IL6R*), altered secretion rates of different sized proteins *(LPA)* and variation in gene copy number *(CCL4).* Blood levels of many of these proteins are correlated with human diseases and the identification of “pQTLs” may in turn help our understanding of disease.

## Introduction

The identification of gene variants that alter the risk of common diseases has proven difficult. Recent genome-wide association studies of disease cases and controls have improved this situation but have shown that, with a few exceptions, most genetic effects on common disease are likely to be small [1].

One successful complementary approach to studying gene-disease associations is to study associations between genetic variation and gene expression. Several genome-wide studies have shown that genetic variation influences gene expression [2]–[8]. Most of these gene regions or variants are found in or close to the gene that codes for the mRNA product (*cis* effects), whilst others are found elsewhere in the genome (*trans* effects). The identification of these effects on gene expression may help understand disease aetiology. However, these data are limited by the fact that they assess gene expression, usually from a single cell type, rather than protein levels, which are likely to be more directly implicated in disease processes [9].

There are no genome-wide analyses of the role of human genetic variation on large numbers of proteins. One way of testing this, and a way that could be relevant to the understanding of human diseases, is *in vivo* studies of serum and plasma levels of proteins. There are likely to be many factors that influence serum and plasma protein levels, only one of which is genetic DNA variation leading to differences in mRNA transcription and subsequent mRNA translation to protein. Other mechanisms could include epigenetic factors, stochastic factors, environmental factors influencing regulation of expression, rates of secretion into the blood from the site of synthesis, proteolysis and clearance, and post-translational modifications such as glycosylation.

In this study we tested the hypothesis that common genetic variation influences protein levels in a human population. We used 1200 European individuals from the population based InCHIANTI study[10] with fasting measures of 42 proteins available. The proteins included many implicated in common diseases and conditions including inflammatory cytokines such as interleukins (metabolic and inflammatory conditions), insulin (diabetes), chemokines (e.g. macrophage inflammatory protein beta, implicated in HIV progression to AIDs), adipokines (e.g. adiponectin, leptin, resistin, implicated in metabolic conditions) and liver function markers. Summary details of individuals and traits are given in Table 1 and Table S1.

**Table 1:** Basic characteristics of the InCHIANTI study population.

Characteristic	N	Mean (95% CI) or Percentage
Age (years): Age range	1200	68.4 (67.5–69.3): 21–102
Gender (%female)	1200	55.2%
BMI: BMI range	1131	27.12 (26.87–27.36): 17.99–46.57
Current Smokers (%)	1200	18.80%
Hypertension (via blood pressure tests) (% case)	1176	42.60%
Ever taken drugs for hypertension (current and/or former)	927	38.40%
Diabetes (% case)	1200	11.10%
Myocardial Infarction (% case)	1200	4.00%
Use of Lipid lowering treatment in last 5 years	1167	5.60%
Use of Steroids in last 5 years	1174	8.00%

## Results

We used data from 496,032 single nucleotide polymorphisms (SNPs) from across the autosomal genome with minor allele frequencies >1% and which had passed stringent quality control checks (see methods). These SNPs captured 80.5% and 86.5% of European genetic variation, based on HapMap data with minor allele frequencies >1% and >5% respectively at r^2^>0.8.

**Figure 1:**
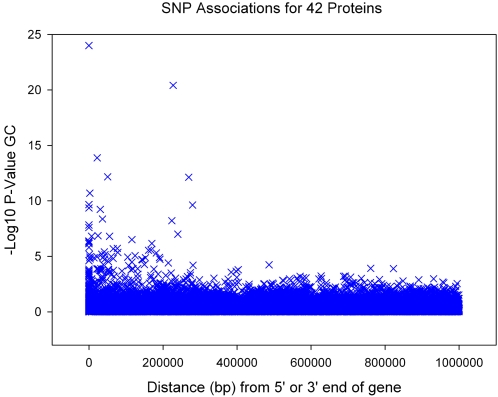
Association of SNPs 1Megabase from each *cis* gene. For each SNP the X axis represents the distance in base pairs from either the 5′ or 3′ end of the gene. If SNPs occur within the gene, either in introns or exons, they are given a distance of zero. SNPs in *IL6R* <1×10^−25^ not shown.

**Figure 2:**
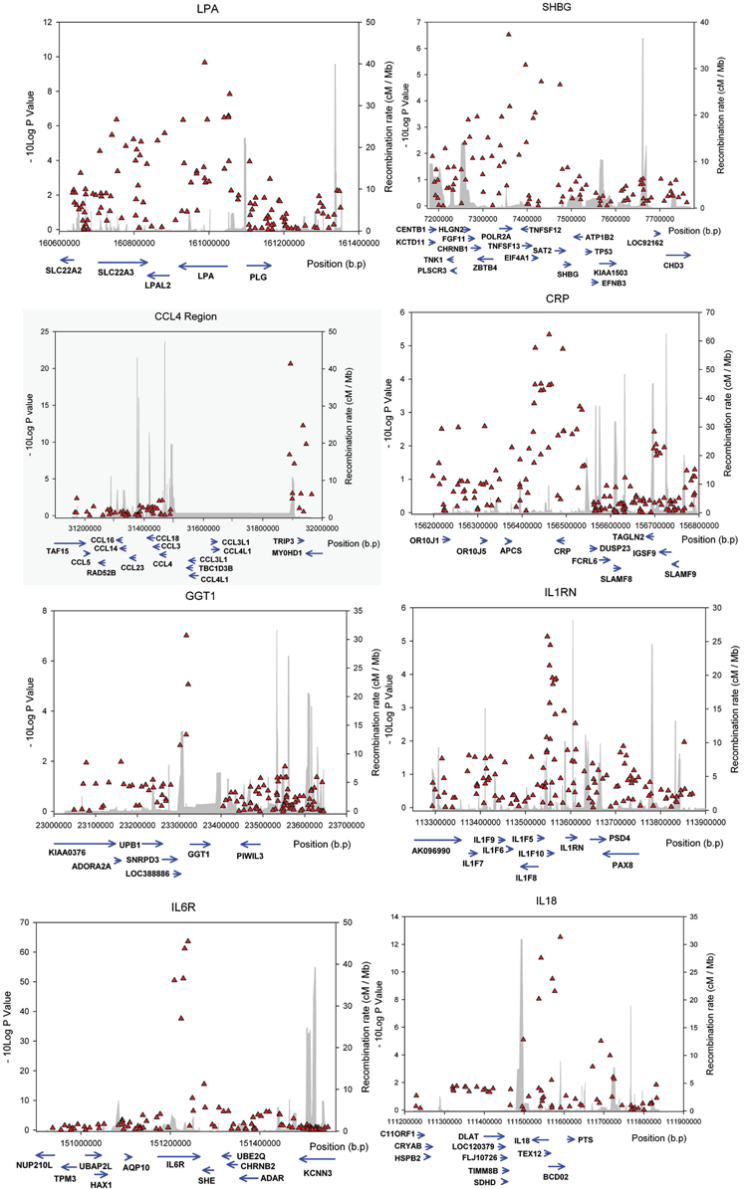
*Cis* genotype effects. X axis shows the distance on the relevant chromosome. Left hand Y axis shows the −log10 p values and right hand Y axis shows the recombination rate as calculated from HapMap data.

We separated our results into *cis* effects and *trans* effects. *Cis* effects were defined as those in the gene(s) coding for the protein or within 300 kb either side of that gene. This was based on a recent study of HapMap variation in relation to gene expression that showed that most cis expression effects occur within this distance of genes [5]. An analysis of all SNPs within a 1Mb window either side of each gene was consistent with this (Figure 1). We used a p value cut off that related to the number of SNPs in or within 300 kb of the gene. If, for example, there were 100 SNPs in a gene region we used 0.05/100 = 0.0005 as significant association. We identified eight cis effects that remained after correction for multiple testing at p<0.05, using 300 kb each side of the relevant gene (Table 2 and Figure 2, Figure S1a). Using 100,000 permutations of the phenotype versus region-wide genotype data confirmed the associations as empirically significant. Given the uncertainty of using 300 kb each side of a gene to define cis effects we repeated these eight analyses using 1Mb of flanking sequence each side of the gene and in each case the association remained (p<0.05).

**Table 2:** Details of *Cis* and *trans* effects.

Protein (units)	Gene	SNP	MAF	Distance (bp)	Mean trait values			GC P	Perm P
					11	12	22		
TNFa (pg/ml)	*ABO*	rs505922	0.34	intron	2.68 (2.53–2.85)	1.66 (1.61–1.72)	1.71 (1.59–1.84)	6.76×10^−40^	<0.0001
IL-6sR (ng/ml)	*IL6R*	rs4129267	0.37	intron	69.92 (66.95–72.99)	100.65 (96.97–104.44)	138.13 (129.94–146.77)	1.82×10^−57^	<0.00001
MIPb (pg/ml)	*CCL4L2*	rs4796217	0.34	227353	74.74 (68.85–81.03)	53.32 (48.64–58.34)	27.21 (21.48–33.83)	3.87×10^−21^	<0.00001
IL18 (ug/ml)	*IL18*	rs2250417	0.44	50476	406.79 (392.66–421.43)	366.58 (355.39–378.12)	330.73 (315.83–346.33)	6.79×10^−13^	<0.00001
LPA (mg/dl)	*LPA*	rs7770628	0.49	intron	0.34/0.18	0.46/0.52	0.20/0.30	4.36×10^−10^	<0.00001
GGT1 (u/l)	*GGT1*	rs5751901	0.39	6917	17.86 (17.11–18.67)	19.6 (18.85–20.41)	21.38 (19.88–23.07)	1.52×10^−7^	0.00076
SHBG (nmol/l)	*SHBG*	rs6761*	0.31	115829	111.67 (106.77–116.77)	100.9 (96.55–105.43)	85.16 (77.65–93.31)	3.08×10^−7^	<0.00001
CRP (ug/ml)	*CRP*	rs12093699	0.29	34092	2.26 (2.07–2.47)	2.74 (2.48–3.03)	3.64 (2.77–4.78)	6.36×10^−6^	0.0038
IL1RA (pg/ml)	*IL1RN*	rs6761276	0.37	43158	118.6 (112.49–125.05)	142.16 (135.73–148.89)	141.23 (126.74–157.37)	7.27×10^−6^	0.00097

MAF = minor allele frequency, SNP = single nucleotide polymorphism and represents the best p value. Distance represents the distance from the gene or location within gene.

GC P represents the p value corrected for inflation factors but not multiple testing. Mean trait values are back transformed values from transformed means, except for LPA where frequencies of genotypes in the low (first value) and high (second value) level groups are given. 11 = common hom, 12 = het, 22 = minor allele homozygote. Perm = Permutation.

P values based on 100,000 region wide (gene +-300 kb) permutations for *cis* effects and 10,000 genome-wide permutations for *trans* effects (“<” indicates the observed p value did not occur in these numbers of permutations). ^*^Further analysis shows that this signal is driven by a SNP, rs1799941, in partial LD with rs6761 – see text.

For three of the eight genes showing cis effects, the associations have been reported in other studies, as part of candidate gene approaches. Variants in or close to the interleukin 6 receptor (IL6R) and C-reactive protein (CRP) genes, are closely correlated with those previously reported [11]–[13](r^2^ 0.96 and 0.91 for IL6R and CRP respectively) and are associated with 0.69 (95%CIs:0.62–0.77), and 0.20 (95%CIs:0.12–0.29) per allele standard deviation differences in their respective protein levels. The SNP in the sex-hormone binding globulin (SHBG) gene, rs6761, was associated with SHBG protein levels with a per-allele effect size of 0.21 (95%CIs:0.13–0.30) standard deviations. This association appeared to be independent of a previously reported variant, rs1799941 [14],[15]. These two SNPs are in moderate linkage disequilibrium (LD) with each other (r^2^ = 0.1) and both remain associated with SHBG levels in the InCHIANTI study when correcting for the presence of the other (p = 0.008 for rs6761 correcting for rs1799941 and p = 0.003 for rs1799941 correcting for rs6761). We therefore genotyped these two variants in an additional 4590 individuals from the WATTs (n = 546) and the The Northern Finland 1966 Birth Cohort (NFBC1966, n = 4044) studies. Details of replication studies are given in Table S2. The association between rs1799941 and SHBG levels replicated (p = 1.4×10^−12^) and meta-analysis of all three studies provided very strong evidence of association (p = 1.8×10^−16^). Conditional analyses using all three studies showed that the association was driven by rs1799941 (p = 1.6×10^−13^ correcting for rs6761) rather than rs6761 (p = 0.38 correcting for rs1799941).

Five of the cis findings have not been reported in other studies, although we recently reported those in the interleukin18 (IL18)[16] and interleukin1 receptor antagonist (IL1RN) [17]genes in the InCHIANTI study as part of candidate gene studies. The effect sizes of the most strongly associated variants in the interleukin18 (IL18) and interleukin1 receptor antagonist (IL1RN) genes were 0.28 (95%CIs:0.20–0.35) and 0.19 (95%CIs:0.11–0.28) per allele SD differences in their respective protein levels. A novel cis association was that in the gamma-glutamyltransferase 1 (GGT1) gene. Each minor allele of rs5751901 was associated with a 0.21 (95%CIs:0.13–0.29) standard deviation increase in GGT1 levels. Other novel cis findings included those in the CCL4 gene cluster with levels of the protein product macrophage inflammatory protein beta (MIP-1beta). Each minor allele of rs4796217 was associated with a 0.49 (95%CIs:0.41–0.32) SD decrease in MIP-1beta levels. The association in the LPA gene resulted in a per allele odds ratio of 1.71 (95%CIs:1.45–2.02) for having LPA levels >14 mg/dl (46% of participants) compared to participants with LPA levels <14 mg/dl. Two further cis findings are worth noting although they did not stand up to all methods of testing. The third strongest association from across the genome with GP130 levels was in the gene, IL6 signal transducer, that encodes the GP130 protein (rs11574783, p = 6.9×10^−6^). A SNP in the parathyroid hormone (PTH) gene region was associated with PTH levels (rs2170436, p = 6.3×10^−5^). Full details of the best cis association for each of the 42 protein levels measured are shown in Table S3a.

We identified one trans effect after a conservative correction for multiple testing based on the number of genome-wide SNPs and phenotypes tested (0.05/(496,032×42) = 2.4×10^−9^) and permutation testing. Specifically, we identified a polymorphism (rs505922) close to the ABO blood group gene, that was very strongly associated with serum TNF-alpha levels (p = 6.76×10^−40^) (Table 2, Figure S1b). Using 100,000 permutations of the phenotype versus genome-wide genotype data confirmed the association as empirically significant. Closer inspection of this region revealed another SNP (rs8176746) independently associated with TNF-alpha levels and haplotypes formed by the two SNPs were correlated (r^2^ = 0.82) with the three alleles that determine the A, B and O alleles of ABO blood group. Separate genotyping of an additional SNP allowed us to accurately recode individuals with their ABO blood group based on a two SNP haplotype (rs8176746 and rs8176719) (Figure S2). Individuals of blood group O (40%) had TNF-alpha levels 0.86 (95%CIs:0.75–0.97) standard deviations (SD) higher than others. This association appears to be assay specific. Using a second TNF-alpha assay, made by a different company (Luminex) resulted in measures of TNF-alpha that were poorly correlated (r = 0.16, Figure S3a) with those from the first assay (R&D systems HSTA00C, ultra-sensitive ELISA), although each was strongly correlated with other inflammatory markers such as C-reactive protein and Interleukin 6 (Figure S3b). There was no association between ABO blood group and the Luminex measure of TNF-alpha (p = 0.26 O blood group vs other blood groups) (Figure S3c) and no association using a third assay (R&D systems HSTA50 ultra-sensitive ELISA) in a separate group of 1620 white individuals from the Health ABC study (p = 0.60, O blood group vs other blood groups). In InCHIANTI there was no strong evidence that rs505922 was associated with any of the other protein markers (p>0.001). Full details of the best trans association for each of the 42 protein levels measured are shown in Table S3b.

Six of the nine associations relate to proteins correlated with inflammatory or metabolic based disease processes so we further tested the robustness of the associations in InCHIANTI when correcting for a number of further covariates, including presence of cardio-vascular disease, diabetes, smoking status and use of steroid anti-inflammatory or lipid-lowering drugs. We also additionally corrected for total protein levels. All associations remained with very similar effect sizes (Table S4).

We next assessed the likely mechanisms of the cis effects. Positions of SNP-protein-level associations relative to genes are shown in Figure 2 and Table S5. For most of the effects, the correlation between SNPs due to linkage disequilibrium does not allow us to draw any conclusions about whether the effects are due to functional variants 5 prime, 3 prime, or within genes. The mechanism of the association between common variation in the IL6R gene and soluble interleukin-6 receptor levels is known: an amino acid substitution Asp358Ala results in differential proteolysis, or “shedding” of the membrane bound to the soluble form of the IL6r protein[18]. The mechanism of the association between common variation in the LPA and CCL4 gene regions and their protein products may be related to copy number variation in these genes. The LPA finding may be due to the previously described association of different numbers of “kringle” repeats that result in different sized proteins [19]–[21], affecting secretion rates from the liver [22]. The MIP-1beta finding may be due to different copy numbers of the CCL4L1 gene. Previous studies have shown that there are copy number variants, in the form of several copies of the CCL3L1 and CCL4L1 genes, in this region and it is possible that the variants we have found are in linkage disequilibrium with copies of the CCL4L1 gene. Copy number variation of the CCL3L1 gene, has been associated with progression from HIV infection to AIDs[23],[24] although the role of CCL4L1 gene variation is not known. For the remaining cis effects one of the most likely mechanisms is that DNA variation alters gene expression which in turn alters protein levels. To look for effects of cis SNPs on gene expression we searched a database of transcript levels of genes in transformed lymphocytes from a recently described genome-wide association study[25]. The SNP associated with GGT1 serum protein levels in our study (rs5751901) was correlated with a SNP that is associated with GGT1 transcript abundance (rs6519519) (p = 2.4×10^−5^) at r^2^ = 0.71. This suggests that the GGT1 association we have seen with protein levels is due to altered transcript levels. There was no evidence that SNPs near the other genes were associated with altered transcript levels (p>0.001), although data were not available from rs1799941.

We next looked more extensively at the publicly available mRNA data [25] to assess the relationship between gene expression in lymphocytes and protein levels. For each of the 42 proteins we looked for any SNPs within 300 kb of the protein coding gene that were associated with transcript levels of that gene above the genome-wide level of statistical significance (LOD>6.08) [25]. For one protein measured in InCHIANTI, IL1beta, there was a *cis* SNP, rs1143627, associated with transcript levels at LOD = 6.1. However, there was no association between this signal and serum protein levels, based on a SNP, rs10169916, in very strong linkage disequilibrium with rs1143627 (r^2^ = 0.96, p value with serum protein levels = 0.54).

## Discussion

Our study shows that the human genetics of serum and plasma protein levels share several features of the genetics of gene expression levels [26]. First, protein levels can be strongly influenced by common genetic variation. This has been shown before for some proteins, notably common null alleles in the enzymes GSTM1 and GSTT1 are associated with a lack of product [27],[28], but our study provides the first systematic, genome-wide assessment of the role of genetic variation on human protein levels. The effect sizes we observe are relatively large (∼0.19 to ∼0.69 SDs per allele) compared to reproducible effects of common variation on other human quantitative traits such as height[29] and body mass index[30]. This does not rule out the presence of weaker effects that did not reach our statistical thresholds. Second, protein quantitative trait loci (pQTLs) can be successfully mapped using a genome-wide association approach, although fine-mapping and functional studies are needed to narrow down the most likely functional variants for most of these traits. Third, there are *cis* effects and these cis effects are often the strongest in the genome. Further studies are needed to investigate the one *trans* finding we identified with TNF-alpha using one assay but not others. We did not find evidence for a fourth feature highlighted by genetic studies of gene expression: we did not find any “multi-*trans*” effects, where gene variants are associated with levels of multiple proteins.

It is likely that there are other *cis* effects that did not reach our cut off for significance. The need to correct p values for the number of tests performed meant that our study was not well powered to detect *cis* effects less than ∼0.22 or ∼0.18 standard deviations per allele for minor allele frequencies 0.1 and 0.5 respectively (based on p = 0.0005). Known variants that did not reach our criteria included those in the *FGB (fibrinogen beta chain*) and *CCL2* genes, known to alter levels of their protein products, fibrinogen[31] and MCP [32], respectively, but which only reached nominal evidence for association in our data. (rs6056 in the *FGB* gene p = 0.051 and rs1024611 in *CCL2* p = 0.02). Additional *trans* effects may also exist but the need to correct for both the genome-wide number of SNPs and number of phenotypes meant that our study would not have detected effects less than ∼0.30 standard deviations per allele (based on p = 2.4×10^−9^). Given that our Bonferroni-based statistical cut-offs are likely to be conservative we also calculated false discovery rates [33]. For all 496,032 tests across 42 phenotypes we estimated that 5%, 10% and 20% of findings will be false discoveries at p values of ∼1×10^−7^, ∼3.×10^−7^ and ∼1.0×10^−6^ respectively.

For one of the eight *cis* findings, the mechanism is known – differential cleavage of bound to unbound receptor caused by an amino acid changing SNP (nsSNP) results in different levels of soluble IL6 receptor [18]. For two other *cis* findings the associations may relate to copy number variants (CNVs). There are reports that different sized LPA proteins, caused by different numbers of kringle repeats, are likely to result in altered secretion rates from the liver into the blood stream [22]. It is also likely that the MIP-beta finding is caused by copy number variation of the *CCL4L1* gene, although further studies are needed to assess the extent of linkage disequilibrium between the *LPA* and *CCL4L1-*region variants we have found and CNVs in these genes. For the remaining *cis* effects, we have found little correlation between SNPs altering gene expression levels in lymphocytes and protein levels, with the exception of the *GGT1* finding. This is perhaps not surprising given the numerous processes that could influence protein levels and is consistent with the observation in yeast experiments that there is considerable variation in the correlation between expression levels and protein abundance[34]–[37]. For many of our findings, the unstimulated cultured lymphocytes used in the gene expression experiment [8] may not be the most relevant tissue to use to equate expression levels with protein levels. For example it may be interesting to determine whether the SNPs we identified are associated with protein levels from stimulated cells, particularly the inflammatory cytokines, which are known to be significantly elevated upon stimulation with, for example, the bacterial membrane antigen lipopolysaccharide [38]. Another possibility is that associations are caused by nsSNPs that alter antibody binding affinity, and therefore the measurement of protein levels. A full re-sequencing effort would be required to rule this possibility out completely but we note that only two nsSNPs, D356N, in *SHBG*, and R1270S in *LPA* are present in dbSNP, and neither of these are strongly (r^2^<0.5) correlated with the most associated SNPs in our study (both are present in HapMap).

The mechanism of the association between ABO blood group and TNF-alpha levels is not known and further work is needed to identify the source of the discrepancy between the associations when different assays are used. The poor correlation between the two TNF-alpha measurements in the same study suggests the two assays are measuring different parts or fractions of the multi-meric TNF-alpha molecule, which can exist in transmembrane form, as a freely circulating protein, or as bound to soluble TNF receptors. Alternatively the association may be caused by cross-reactivity with ABO antigens. If shown in other studies to have a physiological effect the association of ABO blood group with TNF-alpha levels could help the understanding of the mechanisms behind the associations between blood group O and a reduced risk of thrombotic related diseases[39] but increased risk of gastric ulcers[40].

An important implication of our findings is that they may help dissect the causal direction of the associations between protein levels and correlated traits. Serum and plasma concentrations of many proteins change with disease status, ranging from metabolic and cardiovascular diseases to inflammatory and infectious states. Often it is not known whether altered levels of proteins are involved in disease aetiology or are simply a result of the disease process[12],[41]. The identification of genetic variants that alter protein levels may help dissect these relationships. Given the relatively small effects that common gene variants usually have on disease the identification of protein quantitative trait loci (pQTLs) may be a powerful complementary method of improving our understanding of disease.

## Materials and Methods

### Study Participants

#### InCHIANTI Study

The InCHIANTI study is a population based sample that includes 298 individuals of <65 age and 1155 individuals of age ≥65 years. The study design and protocol have been described in detail previously [10]. The data collection started in September 1998 and was completed in March 2000. The INRCA Ethical Committee approved the entire study protocol.

#### Measurement of Proteins

Venipuncture was performed in the morning after a 12-hour fast. Summary details of mean trait values and the numbers of individuals those measures were available in are given in Table 1. Details of the kits used to measure proteins are given in Table S1 along with intra and inter-assay coefficients. These assays were done at the INRCA central laboratory and performed in duplicate and were repeated if the second measure was more than 10% greater or less than the first. The average of the two measures was used in the analyses.

### Genome-Wide Association Analysis

Genome-wide genotyping was performed using the Illumina Infinium HumanHap550 genotyping chip (ver1 and ver3 chips were used). This product assays >555,000 unique SNPs derived primarily from stages I and II of the International Haplotype Map Project (www.HapMap.org). Experiments were performed as per the manufacturers instructions using 750 ng of genomic DNA extracted from whole blood. After processing chips were scanned on Illumina BeadStation scanners. All data were analyzed in BeadStudio (version 3; Illumina), genotype calls were made using the standard cluster files provided by Illumina. Samples were initially assessed for genotype success rate (>98%) and concordance of reported and genotype gender. Nine samples were removed from further analysis due to gender mismatch. Eighty seven samples failed the cut off genotype success rate of 98%; forty eight of these samples were re-purified and successfully genotyped, thus in total 48 samples were removed from further analysis. Manual checking of genotype clusters was performed for all SNPs listed in Table 2.

### Quality Control

We only used DNA samples for which >98% of all SNPs were scored. To estimate the ethnicity of each of the InCHIANTI samples we used the first two principle components from an EIGENSTRAT[42] analysis of a set of 42,048 independent QC-ed SNPs (generated using PLINK's (http://pngu.mgh.harvard.edu/purcell/plink/index.shtml) LD-based SNP pruning function (using parameters –indep-pairwise 200 10 0.1)) that included InCHIANTI and HapMap CEU, JPT+CHB and YRI samples (http://www.HapMap.org). Only SNPs with a MAF >10% in HapMap were used in the analysis. This revealed that all individuals were of European ancestry (Figure S4). The individuals included 20% that were a first degree relative of another person in the study, as calculated from the Identity by descent (IBD) values generated by the Plink “pairwise-IBD” function. We corrected for any over inflation of statistics due to relatedness or residual population admixture by using an inflation factor for each trait, generated using EIGENSTRAT[42] (Table S3).

We only used SNPs that were called in >98% of samples and had minor allele frequencies in our sample of >1%. SNPs deviating appreciably from the expected population distribution (Hardy Weinberg Equilibrium p<1×10^−4^) were also excluded from the analyses. We calculated how well SNPs passing the QC criteria covered common variation in the genome by identifying all European HapMap proxies at r^2^ ≥0.8 for minor allele frequencies (MAF) of 5% and 1%, and then comparing this number to the HapMap count of all autosomal SNPs ≥ the MAF.

### Individual Genotyping in InCHIANTI

A SNP (rs1799941) previously reported to be associated with SHBG levels was not present on the Illumina chip or in HapMap. We therefore genotyped this separately using Taqman probes (Applied Biosystems).

### Statistical Analyses

#### Protein

Many of the proteins were not normally distributed and so we performed appropriate statistical transformations. Where a simple log transformation was not appropriate, we used the STATA version 9 “ladder” command, which searches a subset of the ladder of powers to attempt to detect a simple transformation. Where such simple transformations were not appropriate (i.e. where the distributions were heavily skewed) we considered the STATA “lnskew0” command which performs a log transformation after adding a constant, thus creating a zero-skewness logged variable. Where this transformation was still not appropriate we considered the STATA “bcskew0” command which performs a box-cox power transformation to approximate normality. For proteins identified as significant using these transformations we further tested the robustness of the results by performing a probit transformation: we ranked all individuals for each trait and assigned Z scores corresponding to percentiles in a normal distribution.

For eight proteins there were a small percentage of individuals who had levels below the assay detection limits. In each case there were less than 13 (1%) individuals with levels below detectable limits, except for Macrophage inflammatory protein beta, for which there were 77 individuals below the detectable limits. The values for these individuals for these traits were coded as zero. For a ninth protein, TNF-alpha, there were seven individuals who had levels above the assay detection limits and the values for these individuals were coded at the maximum detectable value 39.4 pgml-1. Non-parametric analyses using quantile regression in Stata v9.0, for MIP-beta and TNF-alpha showed that the highly significant associations observed with these two markers were not affected by the inclusion of individuals with levels out of the assay range.

For six proteins (Interferon-G, Interleukin-10, Interleukin-12, Interleukin-1b, Interleukin-8 and Monocyte Chemoattractant Protein -1) there were >8% of individuals that had levels below the detectable limits. For these we dichotomized traits at the median, or if there was more then 50% below detectable limits, at this point. There was no transformation which made LipoproteinA normally distributed but 14 mg/dl is used as a standard clinical cut off point for high levels and so was used to dichotomise the variable.

#### Genome-Wide Association Statistics

For each autosomal SNP for each of the 36 proteins with levels as quantitative traits, we performed linear regression using PLINK software with age and sex as covariates. This means we tested just one genetic model, an additive model with one degree of freedom. This model tests if the trait alters by equal amounts with each additional allele across the three genotypes. For the six markers dichotomized into high and low values we also performed a single per allele test across genotypes using PLINK (Cochran-Armitage 1df test for trend).

#### Permutation Testing and Quantile Regression

To assess empirical significance of SNPs reaching significance after Bonferroni correction, we used the maxT function in PLINK. Full details are available at http://pngu.mgh.harvard.edu/purcell/plink/index.shtml but briefly each permutation randomly swaps phenotype values between individuals to provide a new dataset sampled under the null hypothesis, but which preserves any correlation between genotypes. The program then compares each observed test statistic against the maximum of all permuted statistics (i.e. over all SNPs) for each single replicate. For the *trans* effect we performed 10,000 permutations across the entire genome and for the *cis* effects we performed 100,000 permutations across the region (“region-wide”) containing the gene and 300 kb each side. This approach meant that permutation tests were not corrected for relatedness but given the relatively small inflation factors for each trait and the fact that the largest permutation p value in Table 2 is 0.0038 this is unlikely to affect the results appreciably. To further check the robustness of our findings we performed non-parametric analyses using quantile regression in Stata v9.0. Three SNPs exceeded the Bonferroni thresholds for significance, rs11574783 with GP130 levels, rs2170436 with parathyroid hormone levels (both *cis*) and rs1880887 with alkaline phosphatase levels (*trans*) but these associations did not remain after either permutation (p>0.05) or non-parametric tests (p>0.05 after multiplication by number of SNPs).

### False Discovery Rates

To assess false discovery rates we calculated the equivalent q statistic as implemented in the “Qvalue” software [33] and using a single file of p values from all 496,032 SNPs for all 42 phenotypes.

### ABO Blood Group Determination

The 3 major ABO blood groups are determined by SNPs in the ABO gene[43]: the O blood group polymorphism (rs8176719) is a G deletion which generates a premature termination codon, and is recessive. B blood group differs from A at 7 nucleotides, including 4 non-synonymous SNPs. There were two independent signals in the ABO gene, associated with TNF-alpha levels (best SNPs were rs8176746 and rs505922). rs8176746 is one of the 4 non-synonymous polymorphisms determining the B group and the A allele, which changes a leucine to methionine amino acid, is found on all B haplotypes. The O blood group deletion polymorphism was not present on the Illumina chip and so to accurately determine ABO blood group, the O deletion polymorphism was typed in the InCHIANTI samples. The deletion was typed using a Taqman end-point PCR custom assay designed by Applied Biosystems. 20ng of DNA was amplified with 1µl of ABsolute QPCR mix containing ROX reference dye (ABgene) and, following 40 cycles of PCR, fluorescence was measured on a Pherastar plate reader and genotypes assigned with Klustercaller software. Haplotypes were constructed using the B blood group SNP (rs8176746) and the recessive deletion polymorphism for O blood group (rs8176719) (Figure S2). Exactly the same methods were used to assign ABO blood groups to the Health ABC samples, except the lack of genome-wide scan data meant we genotyped both rs8176719 and rs8176746 using Applied Biosystems Taqman assays.

### Replication Studies

Summary details of replication studies are given in Table S2. All individuals are of white European ancestry. To replicate the SHBG finding we used baseline data from the Weston Area T3/T4 Study (WATTS) cohort consisting of people on thyroxine replacement, recruited from GP practices in the Bristol and Weston-super-Mare areas in the West of England between March 2000 and June 2002. Further details have been previously published [44]. We also used The Northern Finland 1966 Birth Cohort (NFBC1966), a study of offspring born in the two northern-most provinces of Finland to mothers with expected dates of delivery in 1966[45]. The subjects included in this analysis are from a subset of individuals who had data taken and DNA extracted aged 31 years[46],[47] To replicate the TNF-alpha finding we used baseline data from The Health Aging and Body Composition study, which is an ongoing prospective study designed to investigate the effect of changes in body composition and weight-related health conditions on incident functional limitation. Use of baseline levels of TNF-alpha have been previously reported [48]. In each case, the serum measure was transformed to normality before testing an additive genetic model with age and sex as covariates. Inverse variance meta-analysis as implemented with the “metan” command in STATAv9.0 was used to combine associations from across studies. In each replication study genotyping call rates exceeded 98% and SNPs were in Hardy Weinberg equilibrium (p>0.05).

### Accession Numbers

Accession numbers for proteins are taken from Swissprot (http://www.ebi.ac.uk/swissprot/): SHBG - PO4278, TNFa - PO1375, IL-6sR - P08887, MIPb - P13236, IL18 - Q14116, LPA - P08519, GGT1 - P19440, CRP - P02741, IL1RA - P18510. Accession numbers for genes are taken from Ensembl (http://www.ensembl.org/index.html): ABO - ENSG00000175164, IL6R - ENSG00000160712, CCL4L2 - ENSG00000129277, IL18 - ENSG00000150782, LPA - ENSG00000198670, GGT1 - ENSG00000100031, SHBG - ENSG00000129214, CRP - ENSG00000132693, IL1RN - ENSG00000136689.

## Supporting Information

Figure S1 Plots represent box-plots except for *LPA* where proportions in high and low groups are given. For each genotype the box is bordered at the 25th and 75th percentiles with a median line at the 50th percentile. Horizontal lines joined to the boxes by vertical lines are calculated utilizing the interquartile range (IQR) which is the difference between the first and third quartile values (Q3–Q1). The upper value is the largest data value that is less than or equal to the third quartile plus 1.5 X IQR and the lower adjacent value is the smallest data value that is greater than or equal to the first quartile minus 1.5 X IQR. Values exceeding the upper and lower adjacent values are called outside values and are displayed as markers. a) *Cis* effects b) *Trans* effect.
(0.04 MB DOC)

Figure S2 A) Map of ABO gene from UCSC genome browser, May04, showing positions of the Illumina panel genotyped SNPs (rs8176746 and rs505922) and the functional O blood group polymorphism (rs8176719). b) Linkage disequilibrium (r2) between the 3 SNPs after the O deletion was typed separately in the InCHIANTI samples. c) The four haplotypes formed by the three SNPs shows how rs505922 splits the A blood group allele haplotype. d) Haplotypes formed by rs8176746 and rs8176719 (the B blood group SNP and the recessive deletion polymorphism that defines O blood group, respectively) and how they define ABO phenotype.
(0.13 MB DOC)

Figure S3 Comparison of TNFA results in InCHIANTI. A) Correlations between transformed TNFA levels (log transformation) measured using an ELISA method (R&D systems, HSTA00C) and a LINCOplex method, (Luminex (HADK2-61K-B). B) Correlations between each of the two transformed TNFA measures and three other key proteins, IL6 levels, high sensitivity C reactive protein levels and albumin levels. C) i)–ii)Histograms of raw TNFA measures, iii)–iv)associations with ABO blood group shown as box plots; and v) associations of R&D systems method with ABO blood group showing association is strongest in the one third of individuals with highest TNFA levels.
(0.09 MB DOC)

Figure S4 InCHIANTI and HapMap samples plotted for the first two principal components obtained from multidimensional scaling of a matrix of "identity by state" genotypes. All InCHIANTI samples cluster tightly around the European HapMap samples. INCH = InCHIANTI samples, CEU, JPT+CHB and YRI = European, combined Japanese and Han Chinese and Yoruban samples from HapMap, respectively.
(0.04 MB DOC)

Table S1 Summary details of participants and mean traits. Abbreviations for proteins are included if they are used elsewhere.
(0.10 MB DOC)

Table S2 Details of SHBG and TNF-alpha replication studies.
(0.03 MB DOC)

Table S3 Supplementary Table 3a and 3b Full details of trans and cis effects for 42 proteins. For the nine regions reaching overall significance we include all SNPs in that region that cross the significance threshold. IL = interleukin. 3a *Cis* results for 42 proteins. Details of only the most strongly associated cis SNP for each gene are given, except for the eight reaching significance in which case details of all SNPs in the cis region <0.001 are also given. 3b Trans results. Details of the most strongly associated SNP in the genome wide scan, excluding the gene coding for the protein, plus 600kb of flanking sequence. For TNF-alpha details of all SNPs in the ABO region <0.001 are also given.
(0.42 MB DOC)

Table S4 Associations of the eight cis and one trans finding in InCHIANTI using different covariates and exclusion criteria. MAF = Minor allele frequency. GC P = p values from table 2 in the main paper (corrected for the inflation factor given in supplementary table 2, age and sex). P2 = P values correcting for relatedness using generalized estimating equations, age and sex. P3 = P values correcting for relatedness using generalized estimating equations, age and sex and using a probit-transformed phenotype. P4 = P values correcting for relatedness using generalized estimating equations, age, sex, myocardial infarction, diabetes, being a current smoker, BMI, use of steroids in the last 5 years and use of lipid lowering treatment in the last five years. P5 = P values correcting for relatedness using generalized estimating equations, age, sex, myocardial infarction, diabetes, being a current smoker, BMI, use of steroids in the last 5 years, and use of lipid lowering treatment in the last five years, and additionally the total serum protein. The number of individuals with missing data for this number of covariates was small such that N's for each test were similar, ranging from 1055 to 1195.
(0.04 MB DOC)

Table S5 Positions of the eight genes with significant cis effects based on Jan 07, NCBI 35, dbSNP125, HapMap phase II data release 21a, in relation to the region covered by all HapMap SNPs tagged at r2>0.2 by the most significant cis effect SNP.
(0.04 MB DOC)
