# Supplementary material for: A Genome-Wide Association Study Identifies Protein Quantitative Trait Loci (pQTLs)
Source: PLoS Genet. 2008 May 9;4(5):e1000072. doi: 10.1371/journal.pgen.1000072 (PMC2362067; doi:10.1371/journal.pgen.1000072)
Supplement: Figure S1 — Plots represent box-plots except for LPA where proportions in high and low groups are given. For each genotype the box is bordered at the 25th and 75th percentiles with a median line at the 50th percentile. Horizontal lines joined to the boxes by vertical lines are calculated utilizing the interquartile range (IQR) which is the difference between the first and third quartile values (Q3–Q1). The upper value is the largest data value that is less than or equal to the third quartile plus 1.5 X IQR and the lower adjacent value is the smallest data value that is greater than or equal to the first quartile minus 1.5 X IQR. Values exceeding the upper and lower adjacent values are called outside values and are displayed as markers. a) Cis effects b) Trans effect. (0.04 MB DOC) [file pgen.1000072.s001.doc]

Figure S1a

Figure S1b
