# Supplementary figures and images for: A Genome-Wide Association Study Identifies Protein Quantitative Trait Loci (pQTLs)
Source: PLoS Genet. 2008 May 9;4(5):e1000072. doi: 10.1371/journal.pgen.1000072 (PMC2362067; doi:10.1371/journal.pgen.1000072)

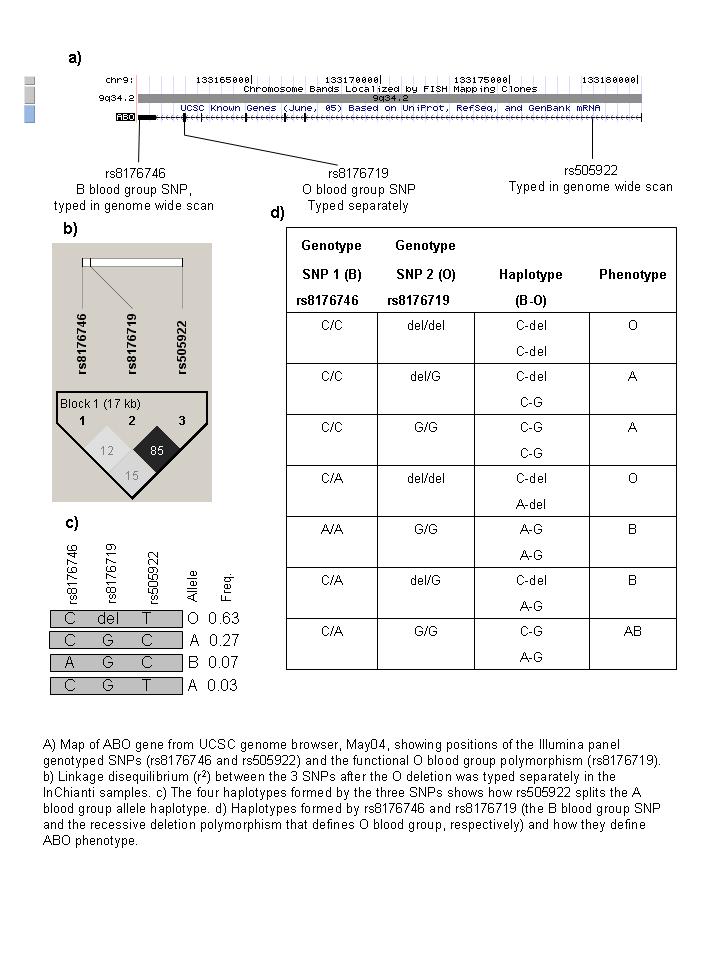

Supplement: Figure S2 — A) Map of ABO gene from UCSC genome browser, May04, showing positions of the Illumina panel genotyped SNPs (rs8176746 and rs505922) and the functional O blood group polymorphism (rs8176719). b) Linkage disequilibrium (r2) between the 3 SNPs after the O deletion was typed separately in the InCHIANTI samples. c) The four haplotypes formed by the three SNPs shows how rs505922 splits the A blood group allele haplotype. d) Haplotypes formed by rs8176746 and rs8176719 (the B blood group SNP and the recessive deletion polymorphism that defines O blood group, respectively) and how they define ABO phenotype. (0.13 MB DOC) [file pgen.1000072.s002.doc]

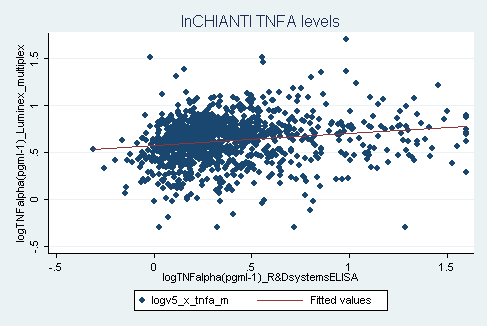
 Figure S3a

r=0.16

t= 5.74

Figure S3b


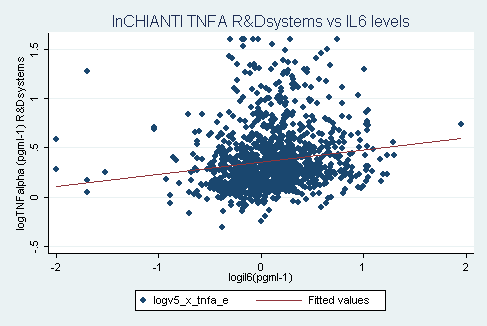

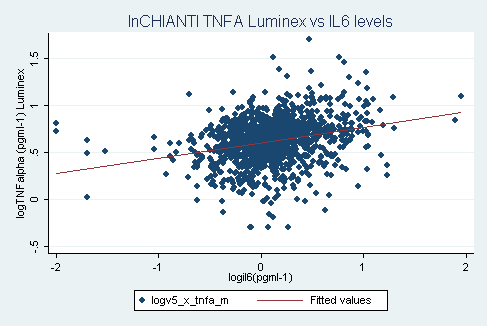


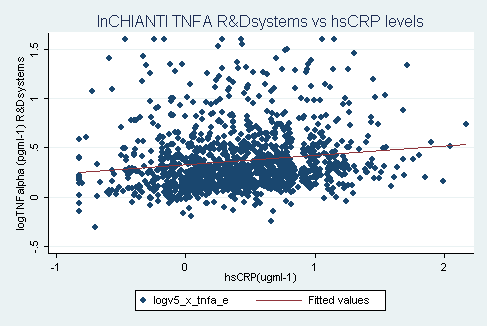

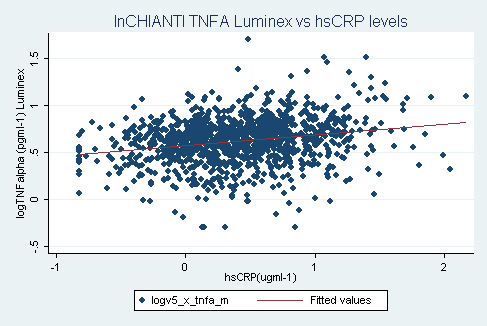


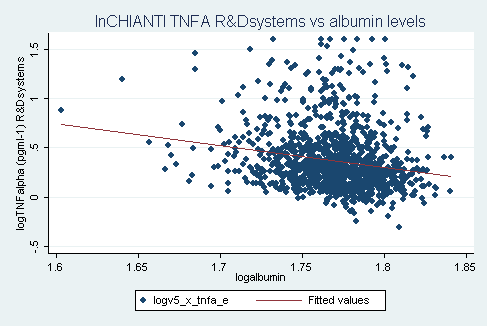

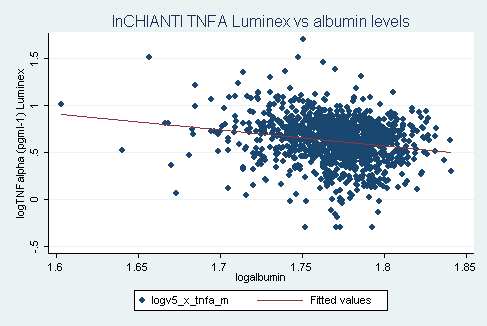


Figure S3c

**
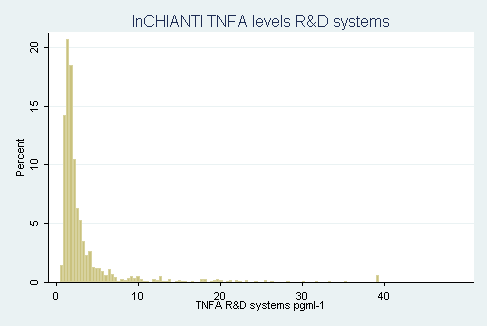

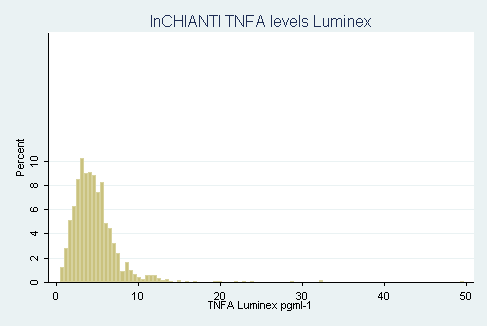
**

p=0.27

p=0.21

p=0.46

p=0.39

p=1x10-9

p=1x10-8

**i)**

**ii)**

p=0.64

**v)**

**
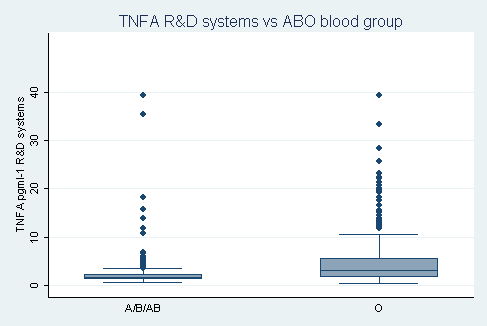

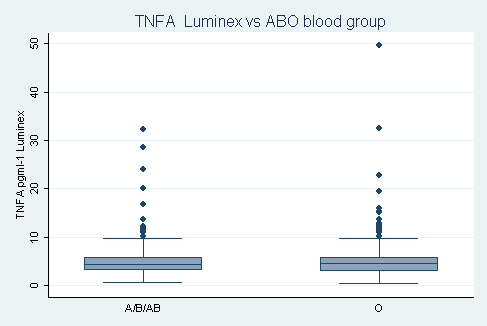
**

p=2x10-44

**iii)**

**iv)**

Supplement: Figure S3 — Comparison of TNFA results in InCHIANTI. A) Correlations between transformed TNFA levels (log transformation) measured using an ELISA method (R&D systems, HSTA00C) and a LINCOplex method, (Luminex (HADK2-61K-B). B) Correlations between each of the two transformed TNFA measures and three other key proteins, IL6 levels, high sensitivity C reactive protein levels and albumin levels. C) i)–ii)Histograms of raw TNFA measures, iii)–iv)associations with ABO blood group shown as box plots; and v) associations of R&D systems method with ABO blood group showing association is strongest in the one third of individuals with highest TNFA levels. (0.09 MB DOC) [file pgen.1000072.s003.doc]
