# Supplementary material for: A Genome-Wide Association Study Identifies Protein Quantitative Trait Loci (pQTLs)
Source: PLoS Genet. 2008 May 9;4(5):e1000072. doi: 10.1371/journal.pgen.1000072 (PMC2362067; doi:10.1371/journal.pgen.1000072)
Supplement: Table S1 — Summary details of participants and mean traits. Abbreviations for proteins are included if they are used elsewhere. (0.10 MB DOC) [file pgen.1000072.s005.doc]

| *Protein* | N | *Sample* | Back Transformed Mean (95% CI) | | *Coefficient of variation* | | *Method used to measure protein* | | | |
| --- | --- | --- | --- | --- | --- | --- | --- | --- | --- | --- |
|  |  |  |  | | *intra-assay* | *inter-assay* |  | | | |
| Adiponectin (ng/l) | 1091 | serum | 11.13  (10.68-11.59) | | <7% | <10% | RIA assay( Human Adiponectin RIA Kit, Linco Research, Inc, Missouri, USA) | | | |
| Albumin (%) | 1195 | serum | 59.24  (59.03-59.44) | | 0.6% | <1% | Percentage of total proteins using agarose electrophoretic technique  (Hydragel Protein (E) 15/30, Sebia, Issy-les-Moulineaux, France). | | | |
| Aldolase (U/l) | 1194 | serum | 2.94 (2.81-3.07) | | Total 17.3% | | Automated test (Roche Diagnostics, GmbH, Mannheim, Germany ) | | | |
| Alkaline Phosphotase (U/l) | 1195 | serum | 194.77  (190.98-198.65) | | 0.5% | 2.2% | Automated colorimetric method (Roche Diagnostics, GmbH,  Mannheim, Germany) | | | |
| Alpha-2 macroglobulin (mg/dl) | 1196 | serum | 194.74  (191.6-197.97) | | <7% | <16% | Immuno-turbidimetric assay (Roche Diagnostics, GmbH, Mannheim, Germany) | | | |
| Alpha-1 globulin (%) | 1195 | serum | 2.6  (2.58-2.63) | | 4.9% | <5% | Agarose electrophoretic technique (Hydragel Protein(E) 15/30, Sebia, Issy-les-Moulineaux, France). | | | |
| C-reactive Protein-hi sensitivity (CRP)(µg/ml) | 1193 | serum | 2.55  (2.39-2.72) | | 5% | | ELISA and colorimetric competitive immunoassay (Roche Diagnostics, GmbH, Mannheim, Germany). | | | |
| Erythropoietin (mU/ml) | 1129 | serum | 9.77  (9.48-10.06) | | <6% | | Advantage erythropoietin chemiluminescence immunoassay  (Nichols Institute Diagnostic, San Clemente, Calif) | | | |
| Fibrinogen (mg/dl) | 1186 | citrate plasma | 351.05  (346.66-355.43) | | 4.1% | | STA Fibrinogen assay (Diagnostic Stago, Roche Diagnostics, France) | | | |
| Free Insulin-Like Growth Factor-1 (pg/ml) | 1048 | serum | 0.53  (0.51-0.55) | | 6.2% | 7.3% | Radio Immuno Assay (RIA) (Diagnostic System Laboratories; Milan, Italy,) | | | |
| Free Triiodothryonine, (Ft3) (pg/ml) | 1137 | EDTA plasma | 4.3  (4.26-4.34) | | <4% |  | Chemiluminescent assay (Vitros FT3 Reagent, Ortho Clinical Diagnostics, Johnson & Johnson Medical S.p.A Section) | | | |
| Free Thyroxine, (Ft4) (ng/dl) | 1142 | EDTA plasma | 1.41  1.39-1.43) | | <3% |  | Chemiluminescent assay (Vitros FT4 Reagent, Ortho Clinical Diagnostics, Johnson & Johnson Medical S.p.A Section) | | | |
| Ferritin (ng/ml) | 1196 | serum | 100.85  (95.41-106.54) | | <7% | | Chemiluminescent immunoassay (Abbot Diagnostics, Rome, Italy). | | | |
| Gamma-Glutamyl Transferase (GGT) (U/l) | 1196 | serum | 19.14  (18.63-19.68) | | 1.5% | 1.3% | Automated enzymatic colorimetric method (Roche Diagnostics, GmbH, Mannheim, Germany) | | | |
| Glutamate Oxaloacetate Transaminase (GOT) (aka Aspartate Transferase) (U/l) | 1196 | serum | 20.15  (19.81-20.5) | | 1.8% | 3.2% | Kinetic method (Roche Diagnostics, GmbH, Mannheim, Germany) | | | |
| Interleukin 6 signal transducer  (GP130) (ng/ml) | 1152 | EDTA plasma | 306.82  (303.16-310.49) | |  | 10% | ELISA (Quantikine Human soluble gp130 immunoassay, R&D Systems, Mineapolis) | | | |
| Glutamic-pyruvic transaminase (GPT) (aka Alanine aminotransferase) (U/l) | 1196 | serum | 18.13  (17.68-18.59) | | 1.6% | 4.4% | Kinetic method (Roche Diagnostics, GmbH, Mannheim, Germany) | | | |
| Haemoglobin (g/dl) | 1189 | whole blood | 13.77  (13.69-13.85) | | not available | | Coulter LH 750 (Beckman Coulter, Instrumentation Laboratory, Milan, Italy)  within 6 h of phlebotomy. | | | |
| Interleukin-18 (µg/ml) | 1193 | serum | 371.11  (363.36-379.04) | |  | 7% | High sensitivity ELISA (Quantikine HS; R&D Systems, Minneapolis, MN) | | | |
| Interleukin-1RA (pg/ml) | 1199 | serum | 132.56  (128.18-137.09) | | < 5% | 7% | ELISA Immunoassay (cytoscreen human IL1RA kit, BioSource Europe S.A., Inc., Camarillo, CA) | | | |
| Interleukin-6 (pg/ml) | 1198 | serum | 1.3  (1.23-1.37) | | Total 7% | | Bio-source cytoscreen ultrasensitivity kits | | | |
| Soluble Interleukin-6 Receptor (sIL-6r) (ng/ml) | 1195 | serum | 91.77  (89.16-94.44) | | <6% | 7% | ELISA Immunoassay (cytoscreen human sIL-6r kit, BioSource International Inc., Camarillo, CA) | | | |
| Insulin (miu/l) | 1038 | EDTA plasma | 9.44  (9.14-9.75) | | <3% |  | Double-antibody, solid-phase radioimmunoassay  (Sorin Biomedica, Milan, Italy). | | | |
| Lactic Dehydrogenase (U/l) | 1196 | serum | 321.67  (318.02-325.37) | | 0.8% | 2.6% | Assessed by an UV assay (Roche Diagnostics, GmbH, Mannheim, Germany) | | | |
| Leptin (ng/ml) | 1116 | serum | 8.27  (7.8-8.78) | | <5% | <7% | ELISA (Human Endocrine LINCOplex Kit, LINCO Research, Inc.,  St. Charles, MO) | | | |
| Myoglobin (ng/ml) | 1185 | serum | 48.47  (47.16-49.8) | | Total <4% | | Two-step immunoenzymatic assay, (Myoglobin Flex reagent cartridge, Dade Behring limited UK) | | | |
| Macrophage inflammatory protein-1b (MIP-1b) (pg/ml) | 1121 | serum | 57.9  (54.45-61.51) | | not available | | Multicytokine detection system (Bio-Rad, Hercules, CA) | | | |
| Parathyroid hormone (pmol/l) | 1154 | EDTA plasma | 20.5  (19.93-21.1) | | <3% | 5.5% | Two-site immunoradiometric assay (N-tact PTHSP, DiaSorin Inc., Stillwater, MN, USA) kit. | | | |
| Resistin (ng/ml) | 1091 | serum | 3.82  (3.73-3.92) | | <4% | <7% | ELISA (ALPCO Diagnostics, Salem, NH, USA) | | | |
| Sex Hormone Binding Globulin (SHBG) (nmol/l) | 1173 | serum | 104.16  (101.06-107.33) | | <7% | <4% | Radioimmunoassay (Diagnostic Pruducts Corporation, Los Angeles, CA) | | | |
| Soluble Transferrin Receptor (mgl/l) | 1175 | serum | 1.25  (1.23-1.28) | | Total <7% | | Chemiluminescent immunoassay (Abbott Diagnostics, Abbott Park, Ill,  and Nichols Institute Diagnostics, San Clemente, Calif). | | | |
| Transforming Growth Factor-b1 (TGF-b1) (pg/ml) | 1191 | serum | 11997.43  (11589.84-12405.03) | | Total <7% | | Highly sensitive quantitative sandwich assays (Quantikine HS, R&D Systems, Minneapolis, MN) | | | |
| Total Insulin-Like Growth Factor-1 (IGF-1) (ng/ml) | 1162 | serum | 118.79  (115.29-122.35) | | <10% | <10% | Immunoradiometric assay (Diagnostic System Laboratories; Milan, Italy. | | | |
| Tumor Necrosis Factor-alpha (TNF-alpha) (pg/ml) | 1187 | serum | 2  (1.94-2.06) | | 7% | <21% | UltraSensitive ELISA (R&D systems, Minneapolis, USA, Quantikine HS HSTA00C) and LINCO*plex* kit, Luminex (HADK2-61K-B) Data given for R&D systems kit. | | | |
| Thyroid Stimulating Hormone (TSH) (miu/l) | 1101 | EDTA plasma | 1.18  (1.11-1.26) | | <5% |  | Chemiluminescent assay (Vitros TSH Reagent, Ortho-Clinical Diagnostics,  Johnson & Johnson Medical S.p.A Section) | | | |
|  | | | | | | | | | | |
| *Proteins dichotomised* | *N (%)*  *< Detectable Limits* | | *N (%)* | | | *Sample* | | *Coefficient of variation* | | *Method used to measure protein* |
|  |  |  | Lowest Group | Highest  Group | |  |  | Intra-assay | Inter-assay |  |
| Interfreon-G (pg/ml) | 857 (76.52) | | 859 (76.56) | 263 (23.44) | | serum | | not available | | Multicytokine detection system (Bio-Rad, Hercules, CA) |
| Interleukin-10 (pg/ml) | 90 (8.0) | | 561 (50.04) | 560 (49.96) | | serum | |  | <9% | ELISA Immunoassay (Human IL10 CytoSETS ^TM^ ELISA kits (Biosource Internetional Inc., Camarillo California USA) |
| Interleukin-12 (pg/ml) | 422 (37.71) | | 561 (50.04) | 560 (49.96) | | serum | | not available | | Multicytokine detection system (Bio-Rad, Hercules, CA, USA) |
| Interleukin-1B (pg/ml) | 655 (58.48) | | 656 (58.47) | 466 (41.53) | | serum | | <7% | <9% | UltrasensitiveELISA Immunoassay (Human IL-1β US, Biosource Internetional Inc., Camarillo California USA) |
| Interleukin-8 (pg/ml) | 508 (45.44) | | 554 (49.42) | 567 (50.58) | | serum | | not available | | Multicytokine detection system (Bio-Rad, Hercules, CA, USA) |
| Lipoprotein A (mg/dl) | 34 (2.87) | | 640 (53.92) | 547 (46.08) | | citrated plasma | | Overall CV 6.6% | | Mercodia Apo(a) ELISA (Uppsala-Sweden) |
| Monocyte Chemoattractant Protein -1 (MCP-1) (pg/ml) | 597 (53.35) | | 598 (53.3) | 524 (46.7) | | serum | | <8% | <21% | Multicytokine detection system (Bio-Rad, Hercules, CA) |
