# Supplementary material for: A Genome-Wide Association Study Identifies Protein Quantitative Trait Loci (pQTLs)
Source: PLoS Genet. 2008 May 9;4(5):e1000072. doi: 10.1371/journal.pgen.1000072 (PMC2362067; doi:10.1371/journal.pgen.1000072)
Supplement: Table S2 — Details of SHBG and TNF-alpha replication studies. (0.03 MB DOC) [file pgen.1000072.s006.doc]

| Replication study | Serum Measure | N (% male) | age (mean, 95%CIs) | Method of protein measurement | Genotyping |
| --- | --- | --- | --- | --- | --- |
| WATTs | SHBG | 546 (16) | 57.2 (56.2 - 58.1) | RIA (Diagnostic Product Corporation, CA) | Kbioscience modified taqman assay |
| NFBC66 | SHBG | 4044 (48) | 31 | Fluoroimmunoassay (Wallac, Inc. Ltd., Finland) | ABI Taqman |
| HealthABC | TNF-alpha | 1768 (52.5) | 73.7 (68.1-79.4) | ELISA (R&D systems, HSTA50) | ABI Taqman |
