# Supplementary material for: A Genome-Wide Association Study Identifies Protein Quantitative Trait Loci (pQTLs)
Source: PLoS Genet. 2008 May 9;4(5):e1000072. doi: 10.1371/journal.pgen.1000072 (PMC2362067; doi:10.1371/journal.pgen.1000072)
Supplement: Table S3 — Supplementary Table 3a and 3b Full details of trans and cis effects for 42 proteins. For the nine regions reaching overall significance we include all SNPs in that region that cross the significance threshold. IL = interleukin. 3a Cis results for 42 proteins. Details of only the most strongly associated cis SNP for each gene are given, except for the eight reaching significance in which case details of all SNPs in the cis region <0.001 are also given. 3b Trans results. Details of the most strongly associated SNP in the genome wide scan, excluding the gene coding for the protein, plus 600kb of flanking sequence. For TNF-alpha details of all SNPs in the ABO region <0.001 are also given. (0.42 MB DOC) [file pgen.1000072.s007.doc]

Table S3a

| **Protein** | **Gene** | **SNP** | **Distance** | **Chr** | **GC P** | **Location** | **Inf** | **Genotypes** | **HWE** | **A1** | **A2** | **MAF** | **Missing** | **Count** | **Cov**  **1%** |
| --- | --- | --- | --- | --- | --- | --- | --- | --- | --- | --- | --- | --- | --- | --- | --- |
| alkaline phosphotase | ALPL | rs10799702 | 13989 | 1 | 0.002224 | 5' | 1.054 | 188/554/452 | 0.39 | G | A | 0.39 | 0.0058 | 172 | 77% |
| TSH | TSHB | rs17479660 | 78417 | 1 | 0.006195 | 3' | 1.03 | 75/432/693 | 0.48 | A | G | 0.24 | 0.0008 | 139 | 65% |
| sIL-6R | IL6R | rs1194589 | 52367 | 1 | 2.94E-05 | 5' | 1.124 | 79/403/717 | 0.04 | A | G | 0.23 | 0.0017 | 106 | 92% |
| sIL-6R | IL6R | rs10908835 | 21188 | 1 | 0.000139 | 5' | 1.124 | 134/544/523 | 0.70 | G | A | 0.34 | 0 | 106 | 92% |
| sIL-6R | IL6R | rs11582424 | 13684 | 1 | 1.33E-05 | 5' | 1.124 | 92/505/602 | 0.36 | C | A | 0.29 | 0.0017 | 106 | 92% |
| sIL-6R | IL6R | rs6684439 | 0 | 1 | 9.32E-46 | - | 1.124 | 165/581/455 | 0.31 | G | A | 0.4 | 0.0008 | 106 | 92% |
| sIL-6R | IL6R | rs4553185 | 0 | 1 | 2.60E-34 | - | 1.124 | 199/604/395 | 0.24 | C | T | 0.42 | 0.0025 | 106 | 92% |
| sIL-6R | IL6R | rs4845623 | 0 | 1 | 2.40E-46 | - | 1.124 | 183/594/423 | 0.31 | T | C | 0.39 | 0 | 106 | 92% |
| sIL-6R | IL6R | rs4537545 | 0 | 1 | 2.60E-55 | - | 1.124 | 178/591/432 | 0.36 | T | C | 0.38 | 0 | 106 | 92% |
| sIL-6R | IL6R | rs4129267 | 0 | 1 | 1.82E-57 | - | 1.124 | 159/582/460 | 0.27 | T | C | 0.37 | 0 | 106 | 92% |
| sIL-6R | IL6R | rs4240872 | 0 | 1 | 2.13E-10 | - | 1.124 | 70/437/693 | 0.94 | C | T | 0.24 | 0.0008 | 106 | 92% |
| sIL-6R | IL6R | rs7514452 | 0 | 1 | 1.53E-06 | - | 1.124 | 38/332/826 | 0.54 | C | T | 0.17 | 0.0042 | 106 | 92% |
| sIL-6R | IL6R | rs12044132 | 22172 | 1 | 1.29E-14 | 3' | 1.124 | 24/277/899 | 0.62 | T | C | 0.14 | 0.0008 | 106 | 92% |
| sIL-6R | IL6R | rs4478801 | 24384 | 1 | 1.38E-07 | 3' | 1.124 | 189/554/454 | 0.36 | G | A | 0.39 | 0.0033 | 106 | 92% |
| sIL-6R | IL6R | rs4584384 | 55509 | 1 | 1.56E-07 | 3' | 1.124 | 189/558/453 | 0.43 | T | C | 0.39 | 0.0008 | 106 | 92% |
| sIL-6R | IL6R | rs7556449 | 65698 | 1 | 2.05E-06 | 3' | 1.124 | 51/363/785 | 0.27 | G | A | 0.19 | 0.0017 | 106 | 92% |
| sIL-6R | IL6R | rs3811448 | 76390 | 1 | 1.90E-06 | 3' | 1.124 | 51/365/785 | 0.31 | A | G | 0.19 | 0 | 106 | 92% |
| sIL-6R | IL6R | rs3766924 | 131638 | 1 | 0.000128 | 3' | 1.124 | 71/456/673 | 0.64 | A | G | 0.25 | 0.0008 | 106 | 92% |
| sIL-6R | IL6R | rs6699825 | 142782 | 1 | 1.95E-05 | 3' | 1.124 | 233/611/355 | 0.32 | G | A | 0.45 | 0.0017 | 106 | 92% |
| sIL-6R | IL6R | rs2335230 | 149440 | 1 | 5.47E-05 | 3' | 1.124 | 67/452/679 | 0.53 | C | A | 0.24 | 0.0025 | 106 | 92% |
| sIL-6R | IL6R | rs884618 | 160969 | 1 | 2.85E-06 | 3' | 1.124 | 186/562/451 | 0.63 | C | T | 0.39 | 0.0017 | 106 | 92% |
| sIL-6R | IL6R | rs9427108 | 167726 | 1 | 2.77E-06 | 3' | 1.124 | 186/563/452 | 0.63 | A | G | 0.39 | 0 | 106 | 92% |
| sIL-6R | IL6R | rs10796927 | 190310 | 1 | 1.27E-05 | 3' | 1.124 | 57/455/688 | 0.11 | C | T | 0.24 | 0.0008 | 106 | 92% |
| sIL-6R | IL6R | rs9426832 | 191698 | 1 | 1.87E-05 | 3' | 1.124 | 224/597/378 | 0.68 | G | A | 0.44 | 0.0017 | 106 | 92% |
| sIL-6R | IL6R | rs6676680 | 222015 | 1 | 0.000315 | 3' | 1.124 | 275/561/350 | 0.09 | A | G | 0.47 | 0.0125 | 106 | 92% |
| CRP | CRP | rs2808660 | 65921 | 1 | 0.000183 | 3' | 1.03 | 50/398/750 | 0.79 | A | G | 0.21 | 0.0025 | 152 | 84% |
| CRP | CRP | rs2592900 | 65451 | 1 | 1.55E-05 | 3' | 1.03 | 63/416/722 | 0.74 | G | A | 0.23 | 0 | 152 | 84% |
| CRP | CRP | rs1446975 | 52738 | 1 | 0.000173 | 3' | 1.03 | 164/538/498 | 0.35 | G | A | 0.36 | 0.0008 | 152 | 84% |
| CRP | CRP | rs12744244 | 34424 | 1 | 0.00019 | 3' | 1.03 | 44/375/756 | 0.85 | A | C | 0.2 | 0.0216 | 152 | 84% |
| CRP | CRP | rs12093699 | 34092 | 1 | 6.36E-06 | 3' | 1.03 | 94/511/596 | 0.30 | A | G | 0.29 | 0 | 152 | 84% |
| CRP | CRP | rs2592887 | 29141 | 1 | 0.00018 | 3' | 1.03 | 199/537/438 | 0.13 | A | G | 0.4 | 0.0225 | 152 | 84% |
| CRP | CRP | rs2794520 | 3264 | 1 | 1.67E-05 | 3' | 1.03 | 127/507/564 | 0.42 | T | C | 0.32 | 0.0025 | 152 | 84% |
| IL10 | IL10 | rs1800896 | 1058 | 1 | 0.003466 | 5' | 1.02 | 176/569/453 | 0.95 | G | A | 0.38 | 0.0025 | 110 | 80% |
| IL1RA | IL1RN | rs6761276 | 43158 | 2 | 7.27E-06 | 5' | 1 | 154/578/466 | 0.24 | T | C | 0.37 | 0.0025 | 131 | 87% |
| IL1RA | IL1RN | rs10186133 | 38526 | 2 | 5.43E-05 | 5' | 1 | 151/572/477 | 0.32 | G | T | 0.36 | 0.0008 | 131 | 87% |
| IL1RA | IL1RN | rs10184259 | 36842 | 2 | 1.32E-05 | 5' | 1 | 97/518/584 | 0.24 | T | C | 0.3 | 0.0017 | 131 | 87% |
| IL1RA | IL1RN | rs13410964 | 32187 | 2 | 0.000127 | 5' | 1 | 262/576/363 | 0.25 | A | G | 0.46 | 0 | 131 | 87% |
| IL1RA | IL1RN | rs4496335 | 30995 | 2 | 0.000195 | 5' | 1 | 254/547/356 | 0.11 | T | C | 0.46 | 0.0366 | 131 | 87% |
| IL1RA | IL1RN | rs4849152 | 26896 | 2 | 0.000147 | 5' | 1 | 63/419/719 | 0.87 | G | A | 0.23 | 0 | 131 | 87% |
| IL1RA | IL1RN | rs1446510 | 23953 | 2 | 0.000134 | 5' | 1 | 261/576/363 | 0.27 | T | C | 0.46 | 0.0008 | 131 | 87% |
| IL1B | IL1B | rs315952 | 295948 | 2 | 0.011292 | 5' | 1.007 | 55/393/738 | 0.79 | C | T | 0.21 | 0.0125 | 124 | 90% |
| Alkaline phosphotase | ALPI | rs10205801 | 272456 | 2 | 0.011001 | 3' | 1.054 | 239/605/356 | 0.56 | G | A | 0.45 | 0.0008 | 91 | 55% |
| IL12 | IL12A | rs11918254 | 36635 | 3 | 0.001422 | 5' | 1.015 | 39/323/825 | 0.30 | T | C | 0.17 | 0.0117 | 109 | 91% |
| Adiponectin | ADIPOQ | rs4012246 | 202132 | 3 | 0.028164 | 3' | 1.027 | 221/583/395 | 0.81 | C | T | 0.43 | 0.0017 | 155 | 69% |
| Soluable transferrin receptor | TFRC | rs11926771 | 119619 | 3 | 0.002321 | 3' | 1.03 | 43/382/776 | 0.71 | A | C | 0.19 | 0 | 89 | 62% |
| IL8 | IL8 | rs1247583 | 117004 | 4 | 0.001344 | 5' | 1.012 | 272/620/302 | 0.18 | A | G | 0.49 | 0.0058 | 71 | 89% |
| Albumin | ALB | rs6855668 | 241495 | 4 | 0.048294 | 3' | 1.07 | 136/549/514 | 0.61 | T | C | 0.34 | 0.0017 | 40 | 74% |
| Fibrinogen | FGB | rs2070016 | 18078 | 4 | 0.01602 | 3' | 1.044 | 28/310/863 | 1 | C | T | 0.15 | 0 | 110 | 81% |
| Fibrinogen | FGG | rs2070016 | 15421 | 4 | 0.01602 | 3' | 1.044 | 28/310/863 | 1 | C | T | 0.15 | 0 | 114 | 86% |
| Fibrinogen | FGA | rs2070016 | 0 | 4 | 0.01602 | - | 1.044 | 28/310/863 | 1 | C | T | 0.15 | 0 | 111 | 77% |
| GP130 | IL6ST | rs11574783 | 689 | 5 | 6.90E-06 | 3' | 1.022 | 9/156/1031 | 0.28 | G | A | 0.07 | 0.0042 | 129 | 85% |
| IL12 | IL12B | rs1363561 | 278873 | 5 | 0.012597 | 3' | 1.015 | 197/577/427 | 0.95 | C | T | 0.4 | 0 | 113 | 70% |
| TNF-alpha | TNFA | rs707928 | 196478 | 6 | 0.015325 | 3' | 1.055 | 116/532/545 | 0.43 | C | T | 0.32 | 0.0067 | 236 | 72% |
| Lipoprotein A | LPA | rs366920 | 214565 | 6 | 3.94E-05 | 3' | 1.035 | 241/587/372 | 0.73 | A | G | 0.45 | 0.0008 | 122 | 83% |
| Lipoprotein A | LPA | rs539298 | 182155 | 6 | 4.91E-06 | 3' | 1.035 | 286/617/295 | 0.33 | A | G | 0.5 | 0.0025 | 122 | 83% |
| Lipoprotein A | LPA | rs9346816 | 169853 | 6 | 6.79E-07 | 3' | 1.035 | 257/618/324 | 0.25 | G | A | 0.47 | 0.0017 | 122 | 83% |
| Lipoprotein A | LPA | rs1510228 | 145112 | 6 | 2.06E-05 | 3' | 1.035 | 66/436/698 | 0.934 | C | T | 0.24 | 0.0008 | 122 | 83% |
| Lipoprotein A | LPA | rs10806731 | 124680 | 6 | 8.52E-06 | 3' | 1.035 | 251/615/335 | 0.32 | G | T | 0.47 | 0 | 122 | 83% |
| Lipoprotein A | LPA | rs7745775 | 119047 | 6 | 0.000134 | 3' | 1.035 | 65/438/697 | 0.81 | G | T | 0.24 | 0.0008 | 122 | 83% |
| Lipoprotein A | LPA | rs2457572 | 116109 | 6 | 3.28E-05 | 3' | 1.035 | 65/437/699 | 0.81 | T | C | 0.24 | 0 | 122 | 83% |
| Lipoprotein A | LPA | rs2504930 | 107296 | 6 | 6.78E-05 | 3' | 1.035 | 53/405/735 | 0.86 | A | G | 0.21 | 0.0067 | 122 | 83% |
| Lipoprotein A | LPA | rs2665357 | 104348 | 6 | 1.17E-05 | 3' | 1.035 | 275/607/307 | 0.49 | G | T | 0.49 | 0.01 | 122 | 83% |
| Lipoprotein A | LPA | rs2048327 | 88983 | 6 | 0.000209 | 3' | 1.035 | 96/501/604 | 0.62 | G | A | 0.29 | 0 | 122 | 83% |
| Lipoprotein A | LPA | rs3127602 | 60274 | 6 | 1.02E-05 | 3' | 1.035 | 97/496/593 | 0.67 | A | G | 0.29 | 0.0125 | 122 | 83% |
| Lipoprotein A | LPA | rs3123630 | 41462 | 6 | 3.89E-06 | 3' | 1.035 | 43/372/785 | 1 | C | T | 0.19 | 0.0008 | 122 | 83% |
| Lipoprotein A | LPA | rs6919346 | 0 | 6 | 7.06E-07 | - | 1.035 | 49/366/786 | 0.46 | T | C | 0.19 | 0 | 122 | 83% |
| Lipoprotein A | LPA | rs11751605 | 0 | 6 | 0.000189 | - | 1.035 | 11/211/973 | 1 | C | T | 0.1 | 0.005 | 122 | 83% |
| Lipoprotein A | LPA | rs7770628 | 0 | 6 | 4.36E-10 | - | 1.035 | 298/588/315 | 0.49 | C | T | 0.49 | 0 | 122 | 83% |
| Lipoprotein A | LPA | rs9365179 | 0 | 6 | 6.78E-07 | - | 1.035 | 163/543/470 | 0.75 | T | C | 0.37 | 0.0208 | 122 | 83% |
| Lipoprotein A | LPA | rs10945682 | 0 | 6 | 5.14E-07 | - | 1.035 | 161/561/470 | 0.80 | A | G | 0.37 | 0.0075 | 122 | 83% |
| Lipoprotein A | LPA | rs1740428 | 0 | 6 | 5.10E-07 | - | 1.035 | 162/545/468 | 0.90 | T | C | 0.37 | 0.0216 | 122 | 83% |
| Lipoprotein A | LPA | rs1321196 | 0 | 6 | 4.09E-07 | - | 1.035 | 163/568/468 | 0.71 | G | A | 0.37 | 0.0017 | 122 | 83% |
| Lipoprotein A | LPA | rs1367211 | 0 | 6 | 0.000144 | - | 1.035 | 104/487/610 | 0.62 | A | G | 0.29 | 0 | 122 | 83% |
| Lipoprotein A | LPA | rs9346833 | 0 | 6 | 2.50E-08 | - | 1.035 | 204/569/428 | 0.55 | C | T | 0.41 | 0 | 122 | 83% |
| Lipoprotein A | LPA | rs783147 | 52699 | 6 | 0.000149 | 5' | 1.035 | 265/619/316 | 0.27 | T | C | 0.48 | 0.0008 | 122 | 83% |
| IL6 | IL6 | rs10485990 | 288070 | 7 | 0.005448 | 5' | 1.031 | 2/126/1062 | 0.57 | G | A | 0.05 | 0.0092 | 126 | 79% |
| Erythropoeitin | EPO | rs11977607 | 176562 | 7 | 0.048623 | 5' | 1.041 | 12/225/963 | 0.88 | G | T | 0.1 | 0.0008 | 57 | 80% |
| Leptin | LEP | rs6467157 | 220568 | 7 | 0.009382 | 5' | 1.007 | 106/525/568 | 0.34 | C | T | 0.31 | 0.0017 | 72 | 67% |
| Ft4 | TG | rs10105992 | 0 | 8 | 0.004137 | - | 1.032 | 268/580/353 | 0.32 | A | G | 0.46 | 0 | 510 | 90% |
| Ft3 | TG | rs2929984 | 123746 | 8 | 0.00656 | 3' | 1.032 | 54/352/772 | 0.10 | A | G | 0.2 | 0.0192 | 510 | 90% |
| GPT (ALT) | GPT | rs10448143 | 56041 | 8 | 0.021221 | 5' | 1.008 | 53/391/724 | 1 | T | C | 0.21 | 0.0275 | 44 | 64% |
| Aldolase | ALDOB | rs1323415 | 228353 | 9 | 0.004491 | 5' | 1.036 | 186/540/466 | 0.16 | T | C | 0.38 | 0.0075 | 158 | 73% |
| Alpha-1 globulin | AMBP | rs7024508 | 144595 | 9 | 0.003617 | 5' | 1.034 | 20/189/945 | 0.01 | C | T | 0.1 | 0.0391 | 224 | 83% |
| GOT (AST) | GOT1 | rs11190179 | 174967 | 10 | 0.00119 | 5' | 1 | 20/238/938 | 0.26 | A | G | 0.12 | 0.0042 | 106 | 80% |
| Insulin | INS | rs217228 | 166300 | 11 | 0.000815 | 3' | 1.06 | 106/466/617 | 0.20 | T | C | 0.29 | 0.01 | 129 | 71% |
| Haemoglobin | HBD | rs2499958 | 241165 | 11 | 0.029249 | 3' | 1.044 | 290/589/318 | 0.60 | G | A | 0.49 | 0.0033 | 230 | 84% |
| Haemoglobin | HBB | rs2499958 | 233802 | 11 | 0.029249 | 3' | 1.044 | 290/589/318 | 0.60 | G | A | 0.49 | 0.0033 | 234 | 82% |
| Parathyroid hormone | PTH | rs2170436 | 280864 | 11 | 6.30E-05 | 3' | 1.034 | 101/551/549 | 0.03 | G | A | 0.31 | 0 | 87 | 82% |
| Lactic dehydrogenase | LDHA | rs16935005 | 197433 | 11 | 0.004669 | 5' | 1.03 | 5/158/1037 | 1 | A | G | 0.07 | 0.0008 | 93 | 84% |
| Ferritin | FTH1 | rs12420131 | 113144 | 11 | 0.026978 | 5' | 1.038 | 3/64/1134 | 0.08 | C | A | 0.03 | 0 | 110 | 67% |
| IL18 | IL18 | rs11214093 | 21384 | 11 | 1.07E-05 | 3' | 1.032 | 299/578/324 | 0.20 | T | C | 0.49 | 0 | 66 | 84% |
| IL18 | IL18 | rs2043055 | 0 | 11 | 1.47E-08 | - | 1.032 | 195/562/444 | 0.47 | G | A | 0.4 | 0 | 66 | 84% |
| IL18 | IL18 | rs5744222 | 2174 | 11 | 2.01E-11 | 5' | 1.032 | 45/342/803 | 0.28 | A | C | 0.18 | 0.0092 | 66 | 84% |
| IL18 | IL18 | rs7123686 | 30643 | 11 | 5.83E-10 | 5' | 1.032 | 166/540/488 | 0.42 | C | A | 0.37 | 0.0058 | 66 | 84% |
| IL18 | IL18 | rs12420140 | 36454 | 11 | 4.33E-09 | 5' | 1.032 | 48/366/784 | 0.52 | A | G | 0.19 | 0.0025 | 66 | 84% |
| IL18 | IL18 | rs2250417 | 50476 | 11 | 6.79E-13 | 5' | 1.032 | 243/572/381 | 0.29 | G | A | 0.44 | 0.0042 | 66 | 84% |
| IL18 | IL18 | rs2091289 | 152552 | 11 | 1.35E-05 | 5' | 1.032 | 166/554/481 | 0.76 | C | T | 0.37 | 0 | 66 | 84% |
| IL18 | IL18 | rs2103173 | 175212 | 11 | 0.00014 | 5' | 1.032 | 157/504/503 | 0.09 | G | A | 0.35 | 0.0308 | 66 | 84% |
| Alpha-2 macroglobulin | A2M | rs2889718 | 83161 | 12 | 0.000831 | 5' | 1.091 | 2/109/1084 | 1 | A | G | 0.05 | 0.005 | 143 | 81% |
| Interferon-G | IFN-? | rs3741646 | 222110 | 12 | 0.001585 | 3' | 1.03 | 67/439/690 | 0.87 | T | G | 0.24 | 0.0042 | 190 | 75% |
| Free IGF-1 | IGF1 | rs11111200 | 201753 | 12 | 0.00569 | 3' | 1.031 | 11/192/985 | 0.60 | A | C | 0.09 | 0.0108 | 111 | 69% |
| Total IGF-1 | IGF-1 | rs17542122 | 240901 | 12 | 0.002478 | 5' | 1.041 | 28/253/920 | 0.04 | G | A | 0.13 | 0 | 111 | 69% |
| Haemoglobin | HBA1 | rs11248850 | 63081 | 16 | 0.010019 | 5' | 1.044 | 234/593/372 | 0.95 | A | G | 0.44 | 0.0017 | 65 | 60% |
| Haemoglobin | HBA2 | rs11248850 | 59277 | 16 | 0.010019 | 5' | 1.044 | 234/593/372 | 0.95 | A | G | 0.44 | 0.0017 | 64 | 60% |
| Haemoglobin | HBM | rs11248850 | 52375 | 16 | 0.010019 | 5' | 1.044 | 234/593/372 | 0.95 | A | G | 0.44 | 0.0017 | 64 | 59% |
| Aldolase | ALDOA | rs8047140 | 141503 | 16 | 0.037525 | 5' | 1.036 | 12/241/946 | 0.56 | T | C | 0.11 | 0.0017 | 29 | 84% |
| GOT (AST) | GOT2 | rs7194417 | 270959 | 16 | 0.06814 | 5' | 1 | 57/364/771 | 0.10 | C | T | 0.2 | 0.0075 | 99 | 75% |
| SHBG | SHBG | rs6761 | 115829 | 17 | 3.08E-07 | 5' | 1.003 | 117/516/567 | 1 | C | T | 0.31 | 0.0008 | 98 | 65% |
| SHBG | SHBG | rs9901643 | 113668 | 17 | 0.000165 | 5' | 1.003 | 8/189/994 | 1 | A | G | 0.09 | 0.0083 | 98 | 65% |
| SHBG | SHBG | rs4511593 | 77956 | 17 | 4.39E-06 | 5' | 1.003 | 121/551/529 | 0.22 | C | T | 0.33 | 0 | 98 | 65% |
| SHBG | SHBG | rs4227 | 42315 | 17 | 1.89E-05 | 5' | 1.003 | 80/457/650 | 1 | G | T | 0.26 | 0.0117 | 98 | 65% |
| SHBG | SHBG | rs1799941 | 68 | 17 | 2.45E-05 | 5' | 1.003 | 621/431/80 | 0.65 | G | A | 0.26 | 0.016 | 98 | 65% |
| Aldolase | ALDOC | rs12602520 | 51468 | 17 | 0.015552 | 5' | 1.036 | 12/220/966 | 1 | C | A | 0.1 | 0.0025 | 46 | 61% |
| MCP-1 | CCL2 | rs1476773 | 256889 | 17 | 0.001423 | 3' | 1.001 | 34/325/842 | 0.67 | A | G | 0.16 | 0 | 205 | 85% |
| MIP- 1b | CCL4L2 | rs8067765 | 223713 | 17 | 6.14E-09 | 3' | 1.011 | 7/198/996 | 0.48 | C | T | 0.09 | 0 | 60 | 61% |
| MIP -1b | CCL4L2 | rs4796217 | 227353 | 17 | 3.87E-21 | 3' | 1.011 | 148/529/524 | 0.44 | C | T | 0.34 | 0 | 60 | 61% |
| MIP -1b | CCL4L2 | rs4796221 | 240125 | 17 | 9.95E-08 | 3' | 1.011 | 206/609/385 | 0.19 | A | G | 0.43 | 0.0008 | 60 | 61% |
| MIP -1b | CCL4L2 | rs2242308 | 269465 | 17 | 7.44E-13 | 3' | 1.011 | 13/216/971 | 0.75 | A | G | 0.1 | 0.0008 | 60 | 61% |
| MIP -1b | CCL4L2 | rs2306595 | 279883 | 17 | 2.33E-10 | 3' | 1.011 | 9/161/1031 | 0.30 | C | T | 0.07 | 0 | 60 | 61% |
| Resistin | RETN | rs2431866 | 98101 | 19 | 0.017043 | 5' | 1.038 | 8/138/1055 | 0.14 | T | G | 0.06 | 0 | 105 | 59% |
| TGF-b1 | TGFB1 | rs10409701 | 298783 | 19 | 0.071349 | 3' | 1.011 | 91/412/698 | 0.01 | A | G | 0.25 | 0 | 84 | 81% |
| Ferritin | FTL | rs12979689 | 193654 | 19 | 0.019443 | 3' | 1.038 | 79/474/646 | 0.55 | A | G | 0.26 | 0.0017 | 106 | 72% |
| GGT | GGT1 | rs5751901 | 6917 | 22 | 1.52E-07 | 5' | 1.032 | 176/556/447 | 0.90 | C | T | 0.39 | 0.0183 | 110 | 75% |
| GGT | GGT1 | rs5751902 | 2553 | 22 | 1.19E-05 | 5' | 1.032 | 158/528/510 | 0.25 | T | C | 0.35 | 0.0042 | 110 | 75% |
| Myoglobin | MB | rs743810 | 259832 | 22 | 0.006419 | 3' | 1.004 | 51/435/710 | 0.14 | C | A | 0.22 | 0.0042 | 130 | 83% |

**Distance** Distance from gene (bp)

**Chr** Chromosome

**GC P** Genomic controlled p value

**Location** Type of sequence most strongly associated SNP falls in

**Inf** Inflation factor: represents degree to which statistics are overinflated by relatedness and residual population stratification

**Genotypes** Counts for 3 genotypes

**Hwd** Hardy Wienberg equilibrium p value

**A1** Allele 1

**A2** Allele 2

**MAF** Minor allele frequency

**Missing** % samples missing a genotype call for a SNP

**Count** Number of SNPs in gene and 300kb either side

**Cov 1%** Coverage of gene +- 300kb each side with 1% MAF, based on European HapMap samples

Table S3b

| **Protein** | **SNP** | **Chr** | **Position** | **Gene*** | **Distance from gene (kb)** | **MAF** | **hwd** | **Inf** | **GC P** |
| --- | --- | --- | --- | --- | --- | --- | --- | --- | --- |
| Fibrinogen | rs10874639 | 1 | 102845930 | COL11A1 | 209 | 0.12 | 0.7769 | 1.044 | 3.11E-06 |
| Resistin | rs4950322 | 1 | 144079652 | CHD1L | 87 | 0.21 | 0.93 | 1.038 | 1.92E-07 |
| Haemoglobin | rs4950322 | 1 | 144079652 | CHD1L | 87 | 0.21 | 0.93 | 1.044 | 6.95E-07 |
| IL6 | rs11683229 | 2 | 63508487 | LOC51057 | 0 | 0.15 | 0.2053 | 1.031 | 5.91E-06 |
| Albumin | rs2900976 | 2 | 71870135 | DYSF | 45 | 0.30 | 0.5792 | 1.07 | 1.43E-06 |
| IL10 | rs11695685 | 2 | 79834457 | CTNNA2 | 0 | 0.30 | 0.05574 | 1.02 | 5.39E-07 |
| sIL-6R | rs7577642 | 2 | 85611408 | SH2D6 | 36 | 0.27 | 0.5552 | 1.124 | 6.97E-06 |
| IL8 | rs10191411 | 2 | 149221700 | EPC2 | 15 | 0.32 | 0.1264 | 1.012 | 4.31E-06 |
| GP130 | rs9834373 | 3 | 78581687 | ROBO1 | 147 | 0.17 | 0.298 | 1.022 | 3.97E-06 |
| Leptin | rs169082 | 5 | 169006634 | DOCK2 | 0 | 0.48 | 0.4519 | 1.007 | 1.29E-06 |
| IL18 | rs9461688 | 6 | 31379695 | HLA-C | 35 | 0.31 | 0.1495 | 1.032 | 4.03E-06 |
| GOT (AST) | rs6455128 | 6 | 62755705 | KHDRBS2 | 0 | 0.19 | 0.1896 | 1 | 2.66E-07 |
| GGT | rs4541776 | 6 | 121944568 | GJA1 | 132 | 0.33 | 0.8446 | 1.032 | 1.72E-06 |
| Alpha-2 macroglobulin | rs9402515 | 6 | 133902038 | EYA4 | 10 | 0.09 | 0.03065 | 1.091 | 1.40E-06 |
| Insulin | rs6930337 | 6 | 148829699 | SASH1 | 0 | 0.06 | 0.8008 | 1.06 | 1.43E-06 |
| TGF-b1 | rs1285407 | 7 | 9039628 | NXPH1 | 474 | 0.35 | 0.1276 | 1.011 | 1.48E-06 |
| Myoglobin | rs6472866 | 8 | 75675689 | GDAP1/PI15 | 237/224 | 0.42 | 0.214 | 1.004 | 7.67E-07 |
| Aldolase | rs10092658 | 8 | 131049654 | FAM49B | 0 | 0.07 | 1 | 1.036 | 5.88E-06 |
| MIP-1b | rs2081670 | 9 | 89966785 | GADD45G/DIRAS2 | 516/485 | 0.15 | 1 | 1.011 | 2.64E-06 |
| GPT (ALT) | rs4742971 | 9 | 105734651 | TMEM38B | 118 | 0.11 | 0.001257 | 1.008 | 8.35E-07 |
| TNF-alpha | rs8176749 | 9 | 133160742 | ABO | 0 | 0.08 | 0.8387 | 1.055 | 4.81E-14 |
| TNF-alpha | rs8176746 | 9 | 133160876 | ABO | 0 | 0.07 | 0.8302 | 1.055 | 1.96E-14 |
| TNF-alpha | rs2073828 | 9 | 133166694 | ABO | 0 | 0.41 | 0.5114 | 1.055 | 1.85E-13 |
| TNF-alpha | rs657152 | 9 | 133168819 | ABO | 0 | 0.38 | 0.7129 | 1.055 | 1.12E-39 |
| TNF-alpha | rs500498 | 9 | 133178201 | ABO | 0 | 0.42 | 0.1378 | 1.055 | 2.43E-15 |
| TNF-alpha | rs505922 | 9 | 133178783 | ABO | 0 | 0.34 | 0.6553 | 1.055 | 6.76E-40 |
| TNF-alpha | rs630014 | 9 | 133179276 | ABO | 0 | 0.49 | 0.1491 | 1.055 | 5.37E-17 |
| TNF-alpha | rs11244079 | 9 | 133214080 | ABO | 34 | 0.04 | 0.7185 | 1.055 | 3.18E-09 |
| CRP | rs7076247 | 10 | 18799635 | CACNB2 | 0 | 0.37 | 0.7574 | 1.03 | 6.04E-06 |
| Lipoprotein A | rs1779876 | 10 | 36688046 | FZD8/ANKRD30A | 718/767 | 0.10 | 1 | 1.035 | 3.49E-06 |
| Lactic dehydrogenase | rs2237878 | 11 | 2722858 | KCNQ1 | 0 | 0.07 | 0.4978 | 1.03 | 1.26E-06 |
| MCP-1 | rs3885683 | 11 | 83133355 | DLG2 | 0 | 0.11 | 0.6722 | 1.001 | 8.33E-06 |
| Soluble transferrin receptor | rs7112513 | 11 | 116542571 | PAFAH1B2 | 0 | 0.13 | 0.3787 | 1.03 | 6.47E-09 |
| Total IGF-1 | rs1939992 | 11 | 126314915 | KIRREL3 | 0 | 0.26 | 1 | 1.041 | 1.01E-06 |
| Ferritin | rs17415853 | 12 | 15822072 | EPS8 | 0 | 0.02 | 1 | 1.038 | 6.83E-07 |
| Alkaline phosphatase | rs1880887 | 12 | 40007697 | PDZRN4/CNTN1 | 110/257 | 0.03 | 0.6213 | 1.054 | 1.19E-10 |
| Alpha-1 globulin | rs11065611 | 12 | 109167207 | ATP2A2 | 15 | 0.06 | 0.1236 | 1.034 | 1.44E-07 |
| Erythropoeitin | rs10466868 | 12 | 130464800 | GPR133 | 316 | 0.12 | 0.005892 | 1.041 | 1.18E-06 |
| IL1RA | rs17369571 | 13 | 21385625 | FGF3 | 211 | 0.16 | 0.5074 | 1 | 1.17E-07 |
| IL12 | rs4770433 | 13 | 22801791 | SACS | 0 | 0.40 | 0.7641 | 1.015 | 4.01E-06 |
| Ft4 | rs16957063 | 15 | 40771580 | CDAN1 | 31 | 0.01 | 0.128 | 1.032 | 4.59E-07 |
| Adiponectin | rs11637235 | 15 | 46420445 | DUT | 0 | 0.28 | 0.28 | 1.027 | 2.76E-06 |
| IL1B | rs4889294 | 16 | 79861260 | BCM01 | 0 | 0.47 | 0.8166 | 1.007 | 4.51E-07 |
| Ft3 | rs3848445 | 17 | 14234746 | HS3ST3B1 | 46 | 0.05 | 0.5302 | 1.032 | 8.44E-09 |
| Interferon gamma | rs208015 | 17 | 43607345 | SKAP1 | 0 | 0.09 | 0.3087 | 1.03 | 1.42E-05 |
| Free IGF1 | rs9303029 | 17 | 78002104 | c17orf62 | 0.2 | 0.08 | 0.0987 | 1.031 | 3.50E-07 |
| SHBG | rs9635963 | 18 | 20003613 | OSBPL1A | 0 | 0.18 | 0.4376 | 1.003 | 2.19E-07 |
| Parathyroid hormone | rs2729409 | 18 | 26395686 | DSC3 | 429 | 0.26 | 0.2641 | 1.034 | 2.70E-06 |
| TSH | rs8109578 | 19 | 10074154 | ANGPTL6 | 0 | 0.08 | 0.001111 | 1.03 | 3.81E-07 |

| Chr | Chromosome |  |  |  |  |  |  |  |
| --- | --- | --- | --- | --- | --- | --- | --- | --- |
| Position | Position on chromosome of SNP | | |  |  |  |  |  |
| Gene* | closest gene |  |  |  |  |  |  |  |
| MAF | Minor Allele frequency | | |  |  |  |  |  |
| hwd | Hardy weinberg Equilibrium p value | | |  |  |  |  |  |
| Infl | Inflation factor for phenotype | | |  |  |  |  |  |
| GC P | Genomic controlled p value | | |  |  |  |  |  |
