# Supplementary material for: A Genome-Wide Association Study Identifies Protein Quantitative Trait Loci (pQTLs)
Source: PLoS Genet. 2008 May 9;4(5):e1000072. doi: 10.1371/journal.pgen.1000072 (PMC2362067; doi:10.1371/journal.pgen.1000072)
Supplement: Table S4 — Associations of the eight cis and one trans finding in InCHIANTI using different covariates and exclusion criteria. MAF = Minor allele frequency. GC P = p values from table 2 in the main paper (corrected for the inflation factor given in supplementary table 2, age and sex). P2 = P values correcting for relatedness using generalized estimating equations, age and sex. P3 = P values correcting for relatedness using generalized estimating equations, age and sex and using a probit-transformed phenotype. P4 = P values correcting for relatedness using generalized estimating equations, age, sex, myocardial infarction, diabetes, being a current smoker, BMI, use of steroids in the last 5 years and use of lipid lowering treatment in the last five years. P5 = P values correcting for relatedness using generalized estimating equations, age, sex, myocardial infarction, diabetes, being a current smoker, BMI, use of steroids in the last 5 years, and use of lipid lowering treatment in the last five years, and additionally the total serum protein. The number of individuals with missing data for this number of covariates was small such that N's for each test were similar, ranging from 1055 to 1195. (0.04 MB DOC) [file pgen.1000072.s008.doc]

| Protein (units) | Gene | SNP | MAF | GC P | P2 | P3 | P4 | P5 |
| --- | --- | --- | --- | --- | --- | --- | --- | --- |
| TNFa (pg/ml) | *ABO* | rs505922 | 0.34 | 6.76 x10^-40^ | 2.55x10^-43^ | 7.91x10^-41^ | 4.89x10^-39^ | 2.53x10^-39^ |
| IL-6sR (ng/ml) | *IL6R* | rs4129267 | 0.37 | 1.82 x10^-57^ | 4.12x10^-71^ | 2.49x10^-76^ | 9.41x10^-67^ | 2.94x10^-69^ |
| MIPb (pg/ml) | *CCL4L2* | rs4796217 | 0.34 | 3.87 x10^-21^ | 3.02x10^-22^ | 1.43x10^-21^ | 1.52x10^-23^ | 2.06x10^-23^ |
| IL18 (ug/ml) | *IL18* | rs2250417 | 0.44 | 6.79 x10^-13^ | 2.14x10^-13^ | 2.88x10^-13^ | 2.95x10^-12^ | 9.33x10^-13^ |
| LPA (mg/dl) | *LPA* | rs7770628 | 0.49 | 4.36 x10^-10^ | 5.03x10^-11^ | N/A | 1.97x10^-9^ | 1.74x10^-9^ |
| GGT1 (u/l) | *GGT1* | rs5751901 | 0.39 | 1.52 x10^-7^ | 2.10x10^-7^ | 1.89x10^-7^ | 7.04x10^-9^ | 8.41x10^-9^ |
| SHBG (nmol/l) | *SHBG* | rs6761 | 0.31 | 3.08 x10^-7^ | 2.47x10^-7^ | 2.34x10^-7^ | 2.47x10^-7^ | 2.61x10^-7^ |
| CRP (ug/ml) | *CRP* | rs12093699 | 0.29 | 6.36x10^-6^ | 7.48x10^-6^ | 2.05x10^-5^ | 7.48x10^-6^ | 9.44x10^-6^ |
| IL1RA (pg/ml) | *IL1RN* | rs6761276 | 0.37 | 7.27x10^-6^ | 6.19x10^-6^ | 1.01x10^-6^ | 8.6x10^-6^ | 7.83x10^-6^ |
