# Supplementary material for: A Genome-Wide Association Study Identifies Protein Quantitative Trait Loci (pQTLs)
Source: PLoS Genet. 2008 May 9;4(5):e1000072. doi: 10.1371/journal.pgen.1000072 (PMC2362067; doi:10.1371/journal.pgen.1000072)
Supplement: Table S5 — Positions of the eight genes with significant cis effects based on Jan 07, NCBI 35, dbSNP125, HapMap phase II data release 21a, in relation to the region covered by all HapMap SNPs tagged at r2>0.2 by the most significant cis effect SNP. (0.04 MB DOC) [file pgen.1000072.s009.doc]

|  |  |  |  |  |  | Distances covered by r^2^>0.2 |  |
| --- | --- | --- | --- | --- | --- | --- | --- |
| Gene | Gene position | SNP | r^2^ >0.2 region | N HapMap SNPs r^2^>0.8 | total | 5' of gene | 3' of gene |
| IL6R | 151190742-151253261 | rs4129267 | 151213088-151324575 | 34 | 111487 | -22346 | 71314 |
| CCL4L2 | 31562580-31564384 | rs4796217 | 31496866-31935416 | 15 | 438550* | 65714 | 371032 |
| IL18 | 111519186-111540050 | rs2250417 | 111497802-111715262 | 49 | 217460 | 175212 | 21384 |
| LPA | 160922926-161055702 | rs7770628 | 160843197-161094182 | 49 | 250985 | 38480 | 79729 |
| GGT1 | 23304271-23349525 | rs5751901 | 23314474-23337900 | 14 | 23426 | -10203 | 11625 |
| SHBG | 7474216-7477395 | rs6761 | 7266847-7477252 | 54 | 210405 | 207369 | -143 |
| CRP | 156495153-156497452 | rs12093699 | 156415943-156542410 | 55 | 126467 | 44959 | 79209 |
| IL1RN | 113591701-113607823 | rs6761276 | 113512897-113633143 | 62 | 120246 | 78804 | 25320 |
