# Supplementary material for: A Genome-Wide Association Study Identifies Protein Quantitative Trait Loci (pQTLs)
Source: PLoS Genet. 2008 May 9;4(5):e1000072. doi: 10.1371/journal.pgen.1000072 (PMC2362067; doi:10.1371/journal.pgen.1000072)
Supplement: Supplementary file 8 [file pgen.1000072.s004.doc]

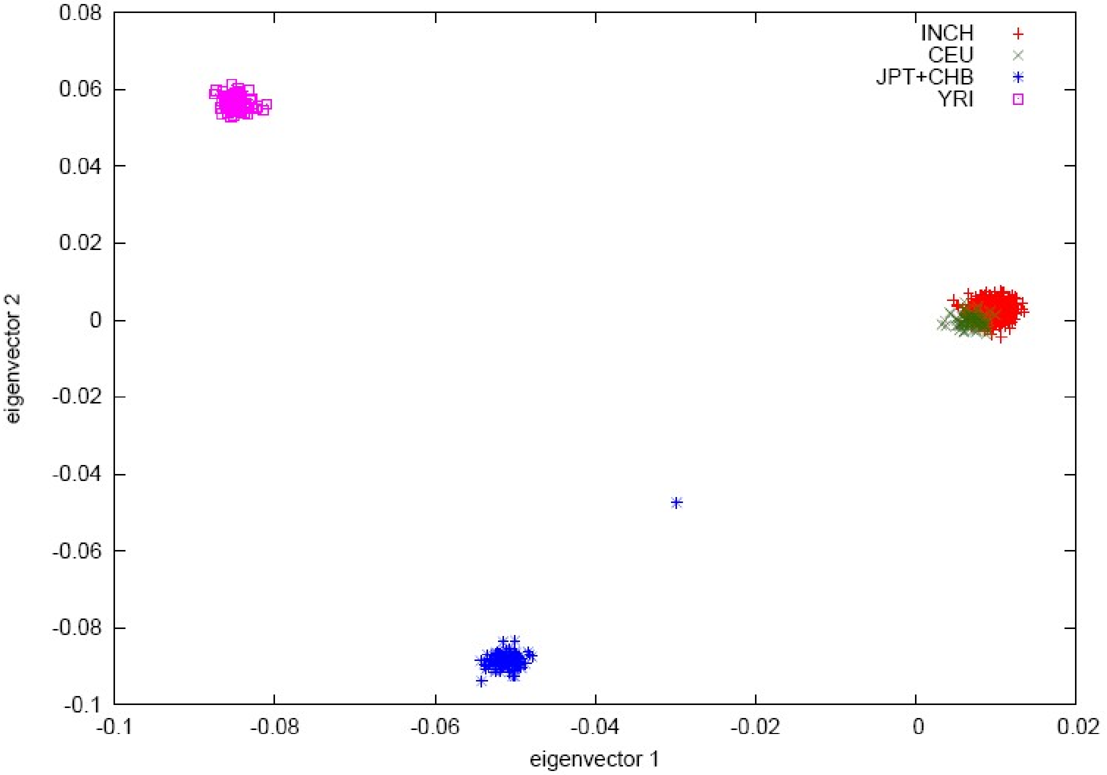

INCH
CEU
JPT+CHB
YRI
eigenvector 2
eigenvector 1
0.08
0.06
0.04
0.02
0
-0.02
-0.04
-0.06
-0.08
-0.1
-0.1
-0.08
-0.06
-0.04
-0.02
0
0.02
